# Open Binding Pose Metadynamics: An Effective Approach for the Ranking of Protein–Ligand Binding Poses

**DOI:** 10.1021/acs.jcim.2c01142

**Published:** 2022-11-19

**Authors:** Dominykas Lukauskis, Marley L. Samways, Simone Aureli, Benjamin P. Cossins, Richard D. Taylor, Francesco Luigi Gervasio

**Affiliations:** † Department of Chemistry, University College London, LondonWC1E 6BT, United Kingdom; ‡ Biomolecular and Pharmaceutical Modelling Group, School of Pharmaceutical Sciences, University of Geneva, CH1211Geneva, Switzerland; § Institute of Pharmaceutical Sciences of Western Switzerland (ISPSO), University of Geneva, CH1211Geneva, Switzerland; ∥ UCB, 216 Bath Road, SloughSL1 3WE, United Kingdom; ⊥ Exscientia Ltd., The Schrödinger Building, Oxford Science Park, OxfordOX4 4GE, United Kingdom

## Abstract

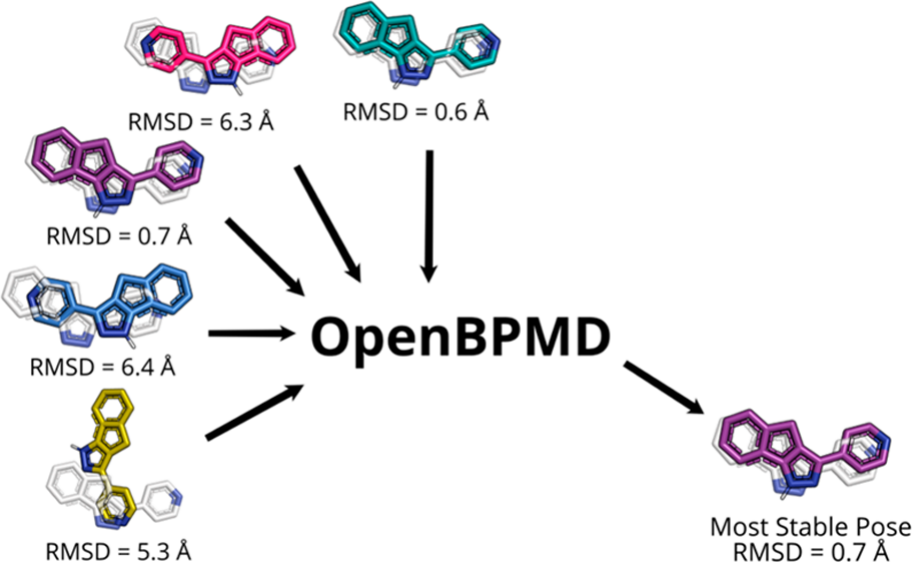

Predicting the correct pose of a ligand binding to a protein and its associated binding affinity is of great importance in computer-aided drug discovery. A number of approaches have been developed to these ends, ranging from the widely used fast molecular docking to the computationally expensive enhanced sampling molecular simulations. In this context, methods such as coarse-grained metadynamics and binding pose metadynamics (BPMD) use simulations with metadynamics biasing to probe the binding affinity without trying to fully converge the binding free energy landscape in order to decrease the computational cost. In BPMD, the metadynamics bias perturbs the ligand away from the initial pose. The resistance of the ligand to this bias is used to calculate a stability score. The method has been shown to be useful in reranking predicted binding poses from docking. Here, we present OpenBPMD, an open-source Python reimplementation and reinterpretation of BPMD. OpenBPMD is powered by the OpenMM simulation engine and uses a revised scoring function. The algorithm was validated by testing it on a wide range of targets and showing that it matches or exceeds the performance of the original BPMD. We also investigated the role of accurate water positioning on the performance of the algorithm and showed how the combination with a grand-canonical Monte Carlo algorithm improves the accuracy of the predictions.

## Introduction

The knowledge of the three-dimensional structure(s) of a protein–ligand complex and the determinants of its thermodynamic stability are fundamental ingredients for rational drug design, both for the hit-discovery phase and the subsequent lead-optimization phase. From the computational perspective, methods such as protein–ligand docking^1−4^ are widely used to address the two related subproblems, finding the most favorable configuration of the small molecule in the target protein (pose generation) and evaluating the stability of intermolecular complexes created during the pose generation phase (pose scoring). Since speed is often of the essence, especially in virtual screening campaigns where large libraries need to be screened against a protein target in a short time, docking algorithms usually trade accuracy for speed by using fast pose generation algorithms and approximate pose scoring functions. These approximations inevitably decrease the predictive power of the algorithms, and increasingly more accurate algorithms often based on molecular dynamics simulations are introduced downstream to docking (or instead of) in drug discovery pipelines to increase the number of predicted true positive hit and lead molecules.

In docking, ligands are introduced into a protein site of interest, and then their degrees of freedom (center-of-mass translations, rotations, and free dihedral angles) are sampled to find the optimal configuration according to an energy function approximating the binding free energy. In the case of induced-fit docking, the algorithm also allows for flexibility of nearby protein residues.^3^ Docking generates multiple candidate poses and ranks them in terms of interaction quality using a scoring function. An extensive study has shown that a typical docking program can correctly rank a native pose (root mean square deviation, RMSD, of less than 2 Å from the native pose) as the top ranked one between 40% and 60% of the time.^5^ Interestingly, it will find at least one pose that can be considered as native around 60% to 80% of the time,^5^ indicating that a key issue is the quality of the scoring function. Since docking is typically used to assess large numbers of ligands, docking algorithms have been built with computational efficiency in mind. To this end, it relies on an empirically fitted scoring function or a simplified physical model, often overlooking crucial contributions to the ligand-target binding free energy. To achieve better pose ranking, a feasible approach is to use methods that model the physics of macromolecules more accurately.

Atomistic molecular dynamics (MD) simulations with an explicit solvent model aim to represent most of the relevant factors needed to replicate the behavior of molecules at the nanoscale. The most straightforward use of MD in binding pose prediction is to simulate the protein and ligand for long enough to observe multiple binding events and then extrapolate the most populated conformation as the true binding mode. Despite reported successes with this method,^6^ the required simulation times (on the order of tens to hundreds of microseconds or more) are far too long to be practical, without extremely specialized hardware.^7^ Another approach to rerank docked poses could be to run shorter unbiased MD simulations of each candidate pose and then rank them according to pose stability, usually measured as the RMSD of the ligand from the pose in question. However, many incorrect poses can be metastable in shorter simulations and may not be reliably distinguished from the native pose.

More or less accurate and expensive enhanced sampling algorithms, such as dynamic undocking (DUck),^8^ coarse metadynamics,^9^ or binding pose metadynamics^10,11^ can help to overcome barriers between such metastable states. Metadynamics (metaD) was designed to accelerate molecular processes of interest by depositing Gaussian-shaped biases along a set of collective variables (CVs) that approximate the reaction coordinate.^12−14^ This method applies a bias to previously observed values of the reaction coordinate, such that the system is “pushed” out of highly populated states into conformations that are observed much less frequently. When the deposited bias is added up, it forms an inverse free energy surface (FES) along the CV, giving the differences between the conformational states and the heights of the barriers separating them. Many molecular phenomena have been successfully studied using metadynamics, including the folding of small proteins, protein conformational dynamics, and ligand binding to proteins.^15−19^

The first use of metadynamics for ligand binding was reported by Gervasio et al.,^18^ where they made use of metadynamics to bias the distance of a ligand from the binding cavity and its orientation in order to explore other metastable states, the unbinding and rebinding paths, and reconstruct the associated free energy profile.

However, this approach requires the definition of system-specific CVs and long sampling times to compute a fully converged free energy landscape associated with the binding, limiting its generalizability. For this reason, various methods have been developed to address these issues, ranging from optimal CVs based on path-like variables,^20−22^ to machine learning,^23−25^ confining boundaries,^17,26−28^ and combination with multiple replica algorithms or more efficient enhanced sampling algorithms.^29,30^ These approaches have been successful, but they are still time-consuming and computationally expensive, making them more suitable for later stages of lead optimization rather than the initial screen of multiple ligands.

Coarse Metadynamics proposed by Masetti et al.^9^ tried to address this issue by using a combination of generalizable CVs to bias docked poses and explore the unbinding path up to the transition state, without trying to fully converge the binding free energy. This approach was based on the observation that the energy barrier for the binding is often similar across different ligands and showed that the local depth of the free energy basins as well as the Δ*G*^‡calc^ even when using only two geometric CVs gives a clear, unambiguous indication of the crystallographic docking geometry and an estimate of the binding affinity of the ligands.

A more recent approach to pose reranking is binding pose metadynamics (BPMD), as proposed by Clark et al. in 2016.^10^ Instead of running long metadynamics simulations until the free energy surface has been fully converged, multiple candidate poses are perturbed in short simulations. These poses are then ranked by stability using the observed RMSD (relative to the initial ligand coordinates) and the persistence of hydrogen bonds during the metaD simulations. More recently, BPMD has been used in conjunction with other approaches, such as water analysis (via WScore),^31^ longer unbiased MD simulations, and relative binding free energy calculations to select native poses.^11^

Here, we present an open-source Python implementation of binding pose metadynamics, called OpenBPMD. BPMD is intended primarily to be used in conjunction with docking to rerank candidate poses in terms of stability. The ligand atoms are subjected to a metadynamics bias, and the ligand pose is given a score according to its stability. OpenBPMD is a Python script that uses the OpenMM molecular dynamics engine^32,33^ to run a metadynamics simulation and MDAnalysis,^34,35^ together with MDTraj,^36^ to process and analyze the simulation in a user-friendly fashion. To validate this implementation, OpenBPMD was applied to the data set used by Clark et al.^10^ obtaining very similar results. We found that OpenBPMD can identify the native pose (RMSD < 2 Å) 88% of the time and that equilibrating the solvent molecules with an advanced water sampling method, based on GCMC/MD, was essential in achieving good results. Our code is open-source and freely available on GitHub (https://github.com/Gervasiolab/OpenBPMD).

## Methods

### Initial Structures

The 3D structures of the protein–ligand poses employed in the present manuscript were obtained from the Supporting Information of the publication by Clark et al.^10^ All systems were prepared through BioSimSpace,^37^ parametrizing the protein and the ligand with the Amber ff14SB^38^ and the GAFF2^39^ force fields, respectively. Ligand partial charges were modeled using the AM1-BCC scheme.^40^ A subset of ligands was reparametrized with RESP partial charges,^41−43^ to test the efficacy of AM1-BCC. This test led to results in agreement between the two systems of partial charges (see Figure S6). The N- and C-termini of the proteins were left uncapped. The protein–ligand complexes were solvated using the TIP3P water model,^44^ and then Na^+^ and/or Cl^–^ ions were added until the systems were charge neutral. BioSimSpace uses “gmx solvate” to set up the solute–solvent boxes.^45^ The OpenMM MD engine was employed to run each simulation.^32,33^ The equilibration involved a potential energy minimization of 10,000 steps (or convergence to the energy tolerance of 10 kJ/mol), followed by 500 ps restrained equilibration in the NVT ensemble with a 2 fs time step. Nonwater heavy atoms were restrained to their initial coordinates during equilibration, with a force constant of 5 kcal/mol/Å^2^. All production simulations used a 4 fs time step with hydrogen mass repartitioning (where the hydrogen mass is set to 4 Da) in the NVT ensemble using a Langevin integrator, with the heat bath reference temperature set to 300 K and the heat bath coupling friction coefficient set to 1 ps^–1^. Periodic boundary conditions were applied, and the particle-mesh-Ewald (PME) method was used to treat long-range electrostatic interactions (cutoff at 10.0 Å).^46^

### Solvation and Equilibration of the Systems

The solvation of the protein–ligand interface can have a great impact on the stability of the complex, e.g., mediating long-range electrostatic interactions. To overcome such an issue, we employed *grand*, a Python module that allows us to perform grand-canonical Monte Carlo (GCMC) sampling of the water molecules during an MD simulation (GCMC/MD).^47−49^ In this way, water molecules may be inserted or deleted within the solvation shell of the ligand, a strategy that has recently been shown to recover water networks seen in crystallographic structures about 70–80% of the time.^50^ The *grand* equilibration process was executed in three stages. The first stage of GCMC/MD is the equilibration of the water distribution. It involves initial 10,000 GCMC moves, followed by 1 ps of GCMC/MD (100 iterations, where each iteration includes 5 MD steps of 2 fs each, followed by 1000 GCMC moves). The second 500 ps NPT simulation was to equilibrate the system volume. The final GCMC/MD stage was to equilibrate the waters at the new system volume and involves 100,000 GCMC moves over 500 ps. To test the influence of water networks on pose stability, solvated poses were simulated with and without this additional *grand* equilibration.

### Enhanced Sampling Simulations

The selected collective variable (CV) was the RMSD of the ligand heavy atoms (using the coordinates at the end of equilibration phase as a reference). The CV also incorporated the anchor atoms (which are used to align the protein, such that the RMSD values are calculated in the same frame of reference, irrespective of protein motion), along with the heavy atoms of the ligand. The selection of the anchor atoms was accomplished according to previously published criteria.^10^ A flat-bottom restraint without a force constant was employed between the anchor atoms and the ligand to fix issues with periodic boundary conditions not being taken into account when OpenMM calculated the RMSD of the ligand during the simulation.

The hill height of the Gaussians is one of the most important parameters in metadynamics. To test the effect of this parameter on the stability of poses, simulations on the entire data set were run twice, using hill heights of 0.3 and 0.05 kcal/mol. An exception is the DPP4 system where only the 0.05 kcal/mol hill height was used throughout, in accordance with the original publication.^10^

A Gaussian width of 0.002 nm was applied on the RMSD CV. The bias potential was deposited every 100 ps, with a bias factor of 4. All OpenBPMD simulations were carried out for 10 ns. The reported OpenBPMD scores are the average score of 10 independent metadynamics runs.

### Scoring Function

Clark et al.^10^ employed two metrics to rank ligand poses in terms of stability, “PoseScore” and “PersScore”. PoseScore is evaluated by computing the RMSD for the heavy atoms of the ligands (obtained by first aligning the simulations on the protein’s secondary structure C_α_ atoms). This metric is particularly efficient in handling significant displacements of ligands’ scaffolds. Nevertheless, it fails to monitor minimal translations of a ligand, even though they may be sufficient to destabilize the protein/ligand interaction network. For this reason, the PersScore metric was selected, to oversee the fraction of long-lasting hydrogen bonds through a metadynamics simulation. However, PersScore is oblivious toward interatomic interactions different from hydrogen bonds, leading to the failure of PersScore for any ligand that does not form hydrogen bonds with the protein.

With the aim of refining the PersScore, we devised a new metric for tracking the persistence of nonbonded interactions between the ligand and the protein during a BPMD simulation: “ContactScore”. Instead of relying only on hydrogen bonds, ContactScore is built on the more generic definition of a “contact” between a ligand and its target, i.e., each couple of heavy atoms belonging to the ligand or the protein within 3.5 Å of each other. In this way, no distinction is made between different kinds of noncovalent interactions, such as π–π stacking, π-halogen interactions, or hydrogen bonds. Procedure-wise, the number of contacts is measured every 100 ps and compared to the amount at the beginning of the OpenBPMD simulation. The final ContactScore is obtained by averaging the number of contacts of the last 2 ns of a simulation.

In order to merge the advantages of the old scoring systems and the new metrics, a composite score was envisioned and named “CompScore” (eq 1):
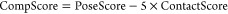
1

**Figure 1:**
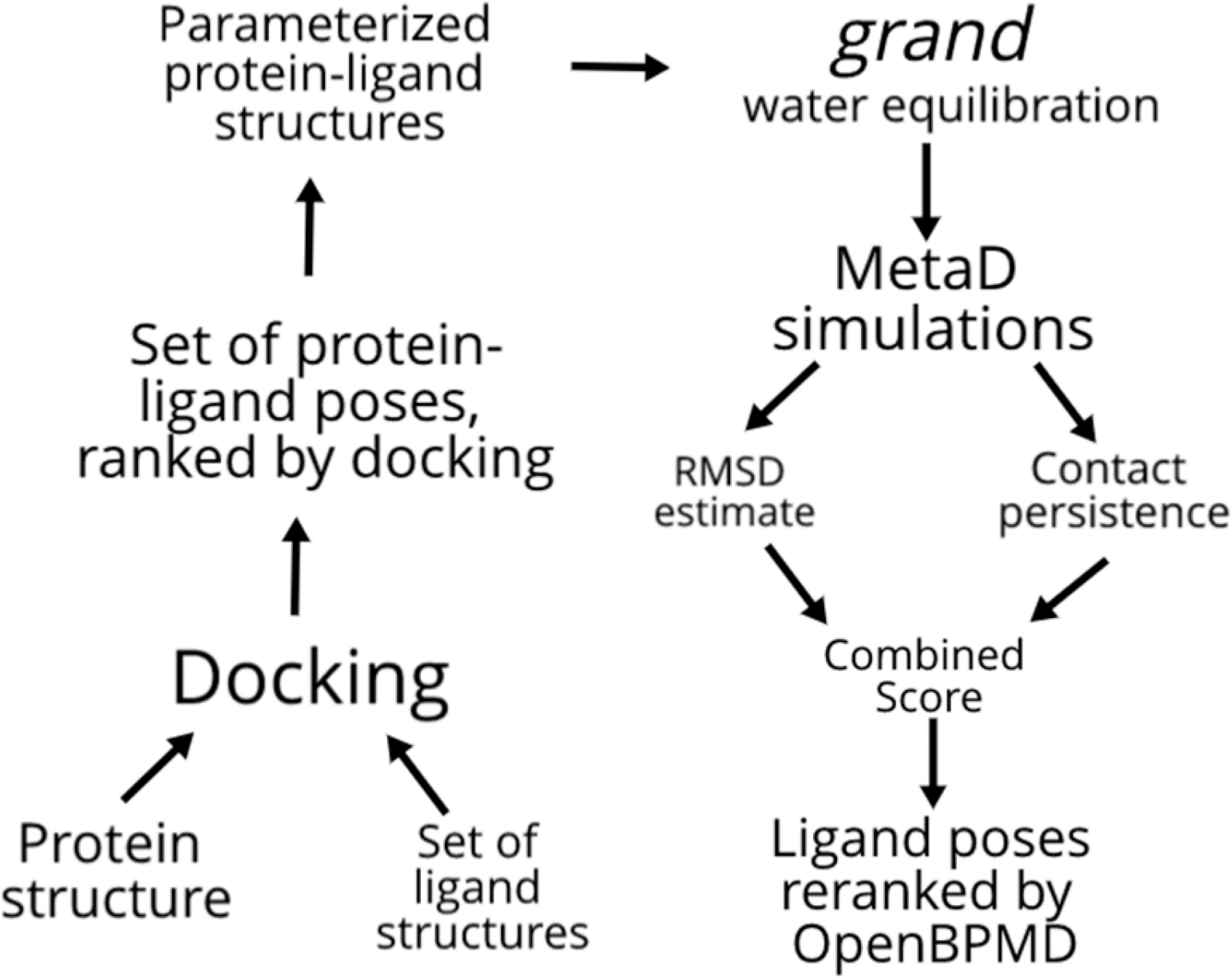
Graphic of the OpenBPMD workflow. The left-hand side part of the workflow can be done with any docking program of choice, and protein–ligand structures can be parametrized using any force field.

This equation is derived from the eq 3 presented in the SI of Clark et al.^10^ in which we replaced the PersScore with the aforementioned ContactScore. Both PoseScore and ContactScore were calculated using MDAnalysis.^34,35^ Simulation trajectories were postprocessed using MDTraj,^36^ to place the solute in the center of the box and account for any periodic boundary conditions. The workflow described above is summarized in Figure 1.

The scripts required to reproduce the results presented in this work are made freely available at https://github.com/Gervasiolab/OpenBPMD.

## Results and Discussion

### BPMD vs OpenBPMD

**Figure 2:**
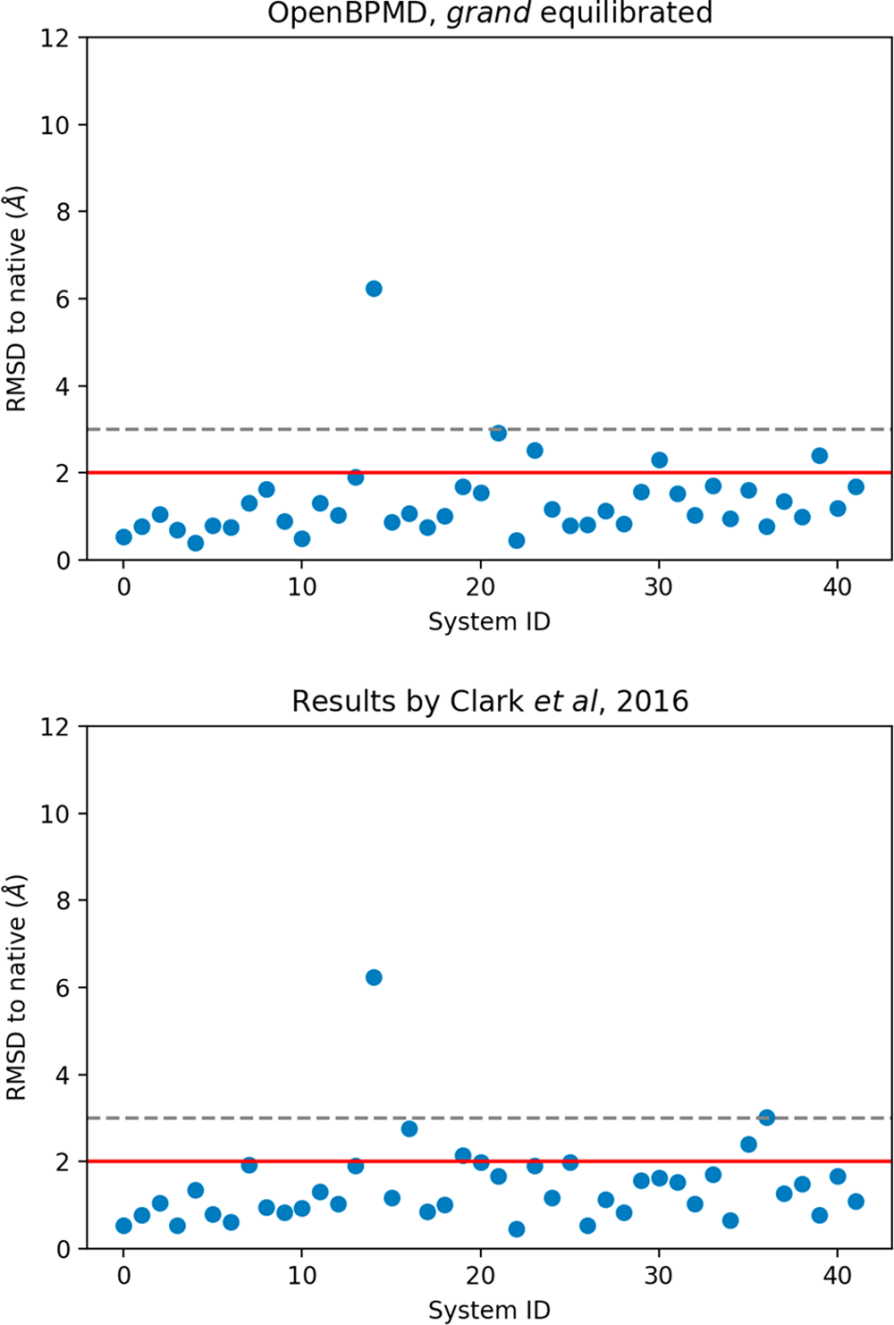
A comparison between the BPMD results obtained using OpenBPMD+*grand* (top) and the previously published results by Clark et al. (bottom). The red and the gray dashed lines demarcate the 2 and 3 Å cutoffs, respectively.

Using an RMSD cutoff of 2 Å for the correct pose classification, with *grand* equilibration and the same hill height of 0.3 kcal/mol for metadynamics, we achieve nearly identical accuracy to Clark et al.^10^ (Figure 2).

Clark et al.^10^ reported that BPMD was able to correctly rank the top pose to be within 2 Å RMSD of the native (crystallographic) pose 88% of the time. OpenBPMD does equally well, correctly finding a low RMSD pose for each ligand with the same success rate. There were two outliers with RMSD > 3 Å for BPMD and one for OpenBPMD. This leads to a top-ranking pose to be within 3 Å RMSD 95% and 98% of the time for BPMD and OpenBPMD, respectively. The pose ranking power of OpenBPMD was also compared to induced-fit docking (IFD). The null hypothesis states that OpenBPMD will not be better at identifying the top pose as within 2 or 3 Å RMSD than IFD, which was correct 64% of the time. As shown in Figure S1, using the two-sided Wilcoxon signed-rank test we can reject the null hypothesis with greater than 95% confidence, i.e., *p* < 0.05. OpenBPMD also displayed a very similar Pearson correlation coefficient between the pose RMSD and the CompScores to the original BPMD implementation^10^ (SI Figure 3).

**Figure 3:**
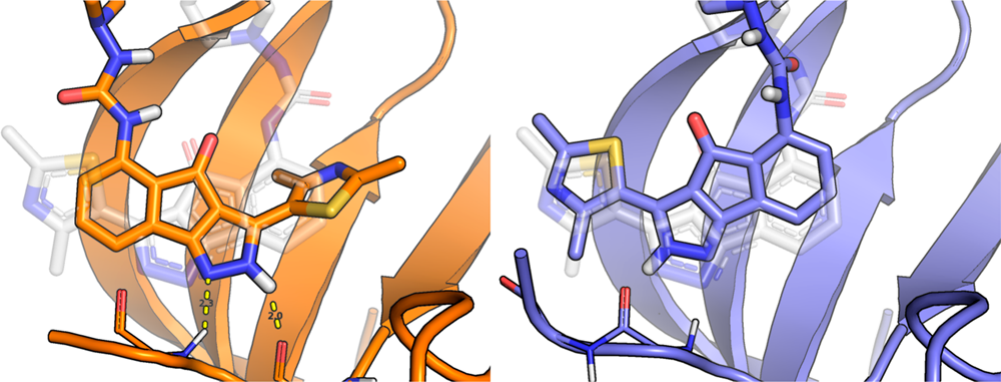
Example low and high RMSD poses for CDK2 ligand D42 (PDB ID: 2b52). Pose 2 on the left (RMSD = 6.24 Å) was predicted to be more stable than Pose 4 on the right (RMSD = 1.39 Å). This is likely owing to the two hydrogen bonds between the pyrazole ring and the backbone of the protein (Leu83 and Glu81). Conversely, such interactions are present in Pose 2 (as indicated with dashed yellow lines). The donor–acceptor atoms in Pose 4 are too far away and at a poor angle for forming a hydrogen bond. The ligand conformation found in the crystal structure is shown in translucent white.

Interestingly, we also identify that the same ligand, D42 from PDB ID 2b52, cross-docked into a CDK2 structure from PDB ID 1wcc, as an outlier. Despite using different force fields and simulation engines, both BPMD and OpenBPMD rank a non-native pose as the most stable one. As mentioned in the Clark et al.,^10^ this is most likely due to a missing set of hydrogen bonds between the ligand and the backbone of the protein (Figure 3). These hydrogen bonds were not formed at the beginning of the simulation and thus not counted toward the persistence score. The same hydrogen bonds were often formed during the metadynamics simulation, pointing out that relying mainly on the initial protein/ligand interactions (i.e., PersScore) may lead to artifacts in the scoring functions.

As a comparison to OpenBPMD, MM-GBSA calculations were run on 10 ns unbiased trajectories using the MMPBSA.py script from Ambertools20.^51,52^ As shown in SI Figure S7, only 60% of the highest affinity poses were correctly ranked as within the 2 Å RMSD cutoff. We also show there is almost no correlation between the MM-GBSA-derived binding affinities and the pose RMSD (SI Figure S7).

### The Need for Advanced Water Equilibration

The initial goal of this work was to follow the protocol of the original BPMD publication,^10^ in order to validate the performance of OpenBPMD against an established data set. The holo complexes were downloaded from the Supporting Information of their publication,^10^ and after preparation using BioSimSpace,^37^ which uses the “gmx solvate” function to set up the simulation boxes,^45^ were evaluated with OpenBPMD. The initial results were disappointing, with rather low success rates of 69% and 71% in ranking the top pose below the 2 Å threshold, using hill heights of 0.3 and 0.05 kcal/mol, respectively (top panels in Figure 5), and a success rate of 86% in both cases, when using an RMSD threshold of 3 Å (bottom panels in Figure 5).

**Figure 4:**
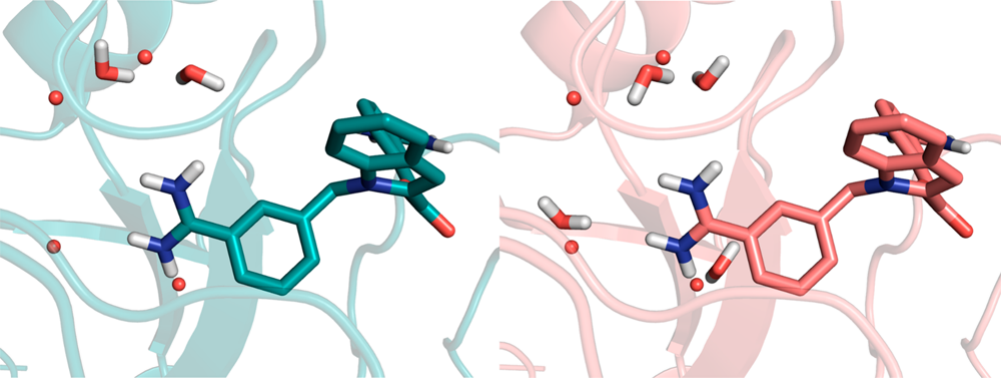
Effect of *grand* equilibration on water positions. Initial water placement (as carried out by the BioSimSpace protocol, see the “Methods” section in the main text) and a short solvent equilibration failed to converge the water networks for a few systems. This figure shows the structure of pose 3 of the FXA ligand CBB found in PDB ID 1lpk docked into a FXA receptor from PDB ID 1g2m before (left) and after (right) *grand* equilibration. The water molecules from the crystal structure are represented by spheres, while the waters from the initial solvation (left) and post*-grand* simulation (right) are shown in sticks. The structure on the left is missing the lower two water molecules, which are present in the crystal and *grand*-equilibrated structures. Without *grand*, pose 3 (RMSD of 1.17 Å from the native pose) was ranked third in stability, while after *grand* it was ranked as the most stable out of the five candidate poses.

While the original protocol included a short solvent equilibration, it did not appear sufficient to sample the bridging waters found buried between the protein and the ligand, owing to the potentially very slow binding kinetics of such waters.^53^ For such an example in this data set, refer to Figure 4. GCMC/MD, a well-tested water equilibration method, was used to address this issue, via the *grand* module.^47−49^ With a thorough *grand* equilibration protocol (described in the Methods section), the OpenBPMD results improved substantially. With a hill height of 0.3 kcal/mol, including *grand* equilibration increased the success rate of OpenBPMD from 69% to 88% (with a threshold of 2 Å). A similar improvement is seen with a hill height of 0.05 kcal/mol, where the success rate increases from 71% to 86%, when using an RMSD threshold of 2 Å. It is not clear why Clark et al.^10^ did not require extensive water equilibration to achieve comparable results. It could be due to differences in the force fields, water models, or how the complexes were set up before any dynamics.

**Figure 5:**
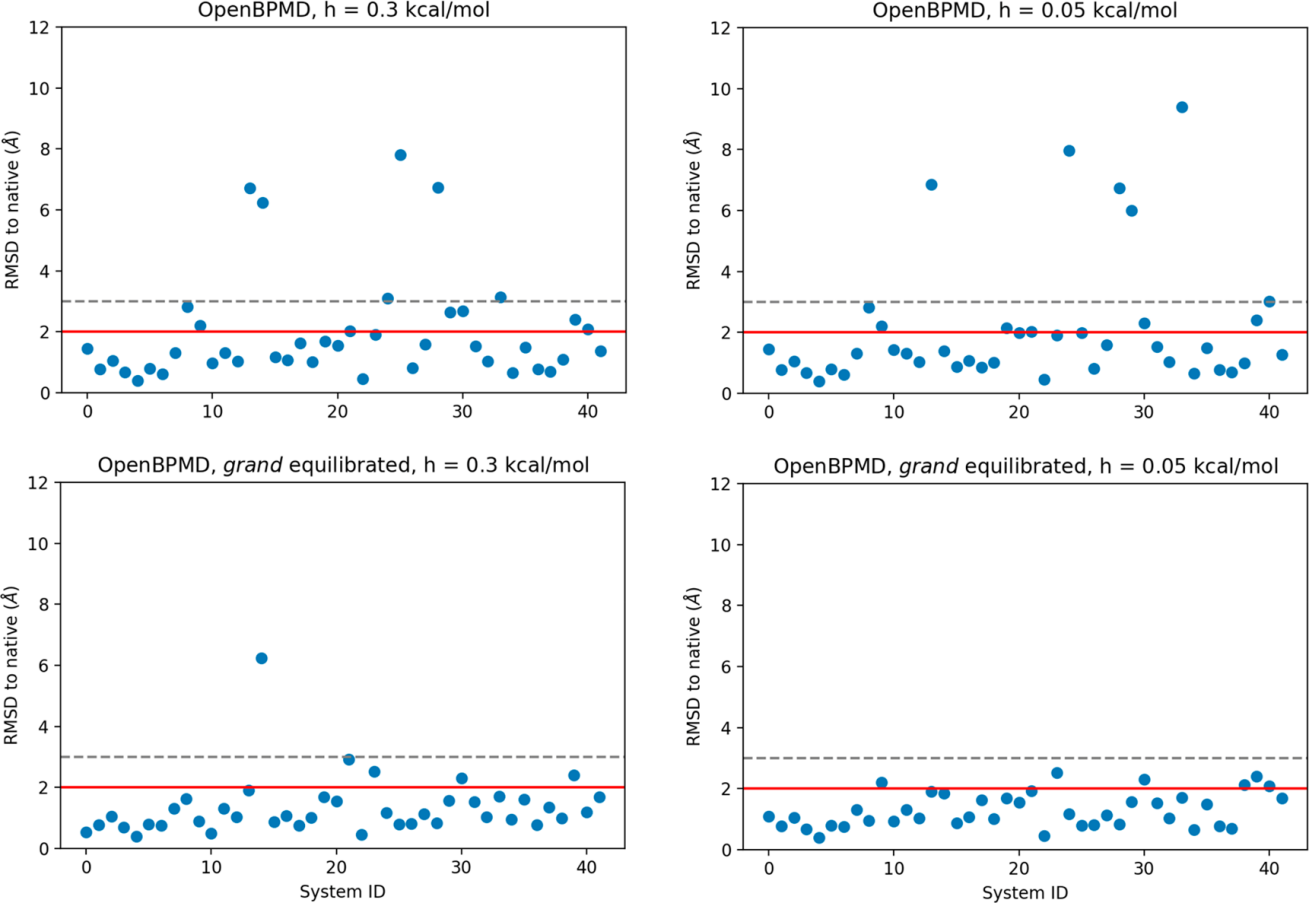
Effects of *grand* equilibration on the accuracy of OpenBPMD scoring. The red and the gray dashed lines demarcate the 2 and 3 Å cutoffs, respectively. Without *grand*, OpenBPMD successfully classified 69% (86%) and 71% (86%) of the poses below the 2 Å (3 Å) threshold, with a hill height of 0.3 and 0.05 kcal/mol, respectively. With *grand*, the success rates increase to 88% (97%) and 86% (100%) of poses correctly classified below the 2 Å (3 Å) threshold, with a hill height of 0.3 and 0.05 kcal/mol, respectively.

### Impact of the Hill Height Parameter

Clark et al.^10^ also tested how the stability scores are affected by different Gaussian hill heights. In short, larger hills will perturb the ligand more. It is important that the bias force is large enough to distinguish between different poses but not so large that the ligands unbind too rapidly. In this work, the effects of a smaller hill height were tested as well. OpenBPMD gives similar results with the 0.05 kcal/mol hill height, with a success rate of 86% below 2 Å RMSD and 100% below 3 Å RMSD. There is some overlap in misclassifications, but this does not appear significant. Three protein–ligand systems were common among the five and six misclassified systems (using the 2 Å threshold) for the 0.3 kcal/mol and the 0.05 kcal/mol setups, respectively.

Notably, the large outlier (where the ligand from the 2b52 PDB structure was docked into the CDK2 receptor from the 1wcc PDB structure), observed in the setup which used *grand* equilibrated structures and a hill height of 0.3 kcal/mol, was no longer an outlier when the smaller hill height was employed. Since the added bias perturbs the system, a larger hill might not be able to distinguish between two poses with similar binding energy, especially if their stability is relatively low. Indeed, in the case of the outlier 2b52, the high RMSD pose had the CompScore of −0.35, while the low RMSD pose showed the CompScore to be −0.66, which is very close. The difference is indeed small, and only with smaller hills (and thus a gentler bias) is OpenBPMD able to distinguish them. This phenomenon has also been reported in the case of the original BPMD paper,^10^ and thus, based on these observations, we advise to use the smaller hills.

### Impact of the Charge Model

A subset of ligands, namely the CDK2 subset, was reparametrized with REST, to compare the effect of using ab initio derived partial charges versus those obtained with the semiempirical AM1-BCC approach. The results are reported in Figure S6. In most cases, the quality of the predicted poses is equivalent. However, for the system that was an outlier (2b52), running OpenBPMD with REST charges and a hills height of 0.3 kcal/mol results in a more accurate pose. Thus, it might be advantageous to use REST charges when possible.

### Performance

OpenBPMD simulations are short, and when run with a high-performance molecular dynamics engine and a 4 fs time step, they can be completed very quickly. For example, a CDK2 protein–ligand system, set up in a triclinic simulation box, typically amounted to around 44000 atoms. On an NVIDIA GTX 2080Ti card, all 10 repeat simulations launched serially would finish in about 5.5 h, running at 430 ns per day. If all 10 simulations were run in parallel, they would complete in around 30 min (Figure S4).

## Conclusions

Here, we have presented the OpenBPMD algorithm developed as an open-source Python module, which allows users to efficiently rerank docked poses. With the addition of an advanced water equilibration method that employs GCMC/MD (via the *grand* software),^47−49^ the protocol presented can successfully predict which pose is within 2 Å RMSD of the crystallographic pose in 88% of the protein–ligand systems investigated. In addition, OpenBPMD displayed a broadly similar correlation between pose RMSD and the composite stability score as reported previously.^10^ With these calculations taking hours on moderate computational resources, we believe OpenBPMD is well suited for integration into many computational pose prediction pipelines.

In this study, OpenBPMD was generally able to correctly rank the top pose as proximal to the native structure; however, the present data set only involved cross-docked poses generated by Glide.^3^ Future work will involve looking at how well OpenBPMD works on poses generated by other docking programs.^1,2,54^ Furthermore, the present data set only involved drug-like molecules. It would be of interest to see how OpenBPMD performs on ranking poses of smaller molecular fragments.

The primary intended use case of OpenBPMD is the reranking of docked poses. However, there are multiple other potential use cases. In structural experiments, the electron densities of ligands are often ambiguous. Sometimes ligands can assume multiple potential conformations that fit the electron density. In these cases, OpenBPMD may assist in the proper assignment of the ligands’ coordinates. Similar investigations have already been done using the proprietary implementation of BPMD.^55^ Much like docking, OpenBPMD could be applied not only for ranking poses but also to carry out virtual screening of compound libraries. Cutrona et al.^56^ showed that metadynamics was able to filter out most of the false positives in a virtual screening campaign. It is possible that pose stability scores have some correlation to ligand binding affinities. Future work will investigate whether OpenBPMD can help enrich libraries, discriminate between decoys and actives, and potentially rank ligands by their affinity.

## Data and Software Availability

The results from the OpenBPMD simulations are included in the three .txt files in the SI. The code that was developed and validated in this project is freely available on GitHub (https://github.com/Gervasiolab/OpenBPMD).

## Supporting Information Available

Results of *grand* equilibrated OpenBPMD simulations with 0.3 kcal/mol hill height (TXT)
Results of *grand* equilibrated OpenBPMD simulations with 0.05 kcal/mol hill height (TXT)
Figure S1, Comparison between IFD and OpenBPMD top-ranked poses; Table S1, Results of OpenBPMD+*grand* simulations on the systems under investigation; Figure S2, PoseScore and ContactScore values for the systems reported in Table S1; Figure S3, Pearson coefficient correlation analysis between the CompScore and the poseRMSD values obtained for 4 different sets of simulations; Figure S4, Performances of OpenBPMD on different hardwares; Figure S5, Atomistic description of the ligand OSC docked into alpha-thrombin; Figure S6, Comparison of OpenBPMD’s pose ranking ability employing different sets of ligands’ partial charges; and Figure S7, MM-GBSA simulations on the docking poses for the systems reported in Table S1. Pose ranking and Pearson correlation analysis (PDF)
Results of MM-GBSA binding affinity calculations for each pose (TXT)

## Abbreviations

BPMD: binding pose metadynamics
CDK2: cyclin dependent kinase 2
DPP4: dipeptidyl peptidase-4
GCMC/MD: grand canonical Monte Carlo/molecular dynamics
IFD: induced fit docking
MM-GBSA: molecular mechanics with generalized Born and surface area solvation
PDB: Protein Data Bank
PKA: protein kinase A
RMSD: root mean square deviation
