# Supplementary material for: Open Binding Pose Metadynamics: An Effective Approach for the Ranking of Protein–Ligand Binding Poses
Source: J Chem Inf Model. 2022 Nov 19;62(23):6209–16. doi: 10.1021/acs.jcim.2c01142 (PMC9749024; doi:10.1021/acs.jcim.2c01142)
Supplement: Supplementary file 3 — ci2c01142_si_003.pdf [file ci2c01142_si_003.pdf]

Supporting Information for:

Open Binding Pose Metadynamics: an effective approach for the ranking of protein-ligand binding poses.

Dominykas Lukauskis,[a] Marley L. Samways,[d] Simone Aureli,[b,c] Benjamin P. Cossins,[d, e] Richard D. Taylor,[d] Francesco Luigi Gervasio[a,b,c,d,]*

a Department of Chemistry, University College London, London WC1E 6BT, United Kingdom

b Biomolecular and Pharmaceutical Modelling Group, School of Pharmaceutical Sciences University of Geneva, CH1211 Geneva, Switzerland

c Institute of Pharmaceutical Sciences of Western Switzerland (ISPSO), University of Geneva, CH1211 Geneva, Switzerland

d UCB, 216 Bath Road, Slough, SL1 3WE, United Kingdom

e Exscientia Ltd., The Schrödinger Building, Oxford Science Park, Oxford OX4 4GE, United Kingdom

*Corresponding author, francesco.gervasio@unige.ch

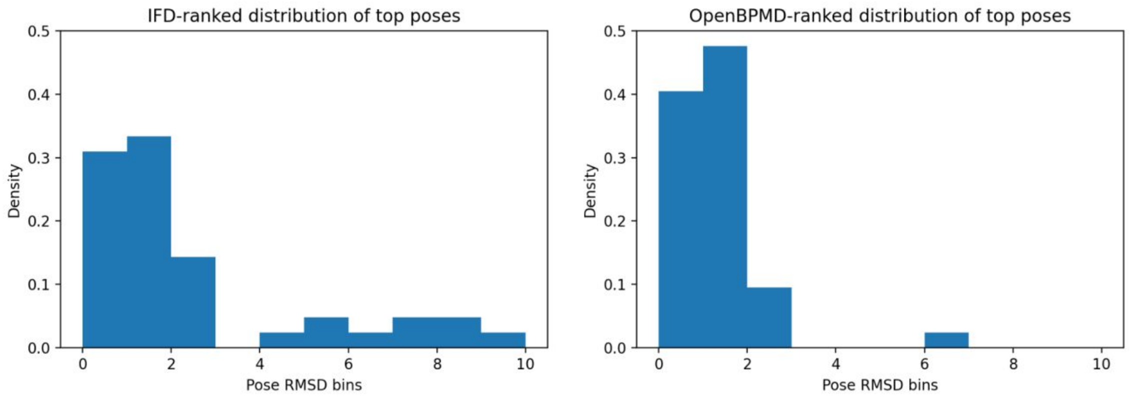

Figure S1. The null hypothesis. On the left, a histogram of top-ranked pose RMSDs determined by induced-fit docking (IFD), on the right, a histogram of the results of the *grand* equilibrated OpenBPMD simulation with a 0.3 kcal/mol hill height. The null hypothesis states that OpenBPMD isn't able to select poses better than IFD. The two-sided Wilcoxon signed-rank test gives the sum of the ranks of the differences above or below zero S = 98.5, with a p value of 0.00197. Using the threshold of p = 0.05, we can reject the null hypothesis.

Table S1. Tabulated results from OpenBPMD+*grand* simulations with 0.3 kcal/mol hill height. The columns with the suffix 'SD' display the standard deviation of each score (i.e., CompScoreSD, PoseScoreSD, and ContactScoreSD), as calculated from 10 independent simulations. As mentioned in the Methods section of the main text, the naming scheme of the OpenBPMD+*grand* simulations is derived from the original publication by Clark *et al.*[1] The names can be decoded as '(protein name)_(protein PDB ID)_(protein name)_(ligand PDB ID)_pose_(pose number)'.

System ID	pose_name	pose_rmsd	CompScore	CompScoreSD	PoseScore	PoseScoreSD	ContactScore	ContactScoreSD
0	alr2_1iei_alr2_2fz8_pose_01	2.14	-1.27	2.16	1.87	0.71	0.63	0.33
	alr2_1iei_alr2_2fz8_pose_02	0.53	-1.68	1.69	1.57	0.71	0.65	0.27
	alr2_1iei_alr2_2fz8_pose_03	1.46	-1.56	2.15	1.8	0.76	0.67	0.34
	alr2_1iei_alr2_2fz8_pose_04	1.1	-0.97	2.09	1.88	0.86	0.57	0.29
	alr2_1iei_alr2_2fz8_pose_05	2.36	-0.61	1.82	2.32	0.98	0.59	0.3
1	alr2_2fzd_alr2_1iei_pose_01	0.78	-2.54	1.25	1.15	0.41	0.74	0.24
	alr2_2fzd_alr2_1iei_pose_02	3.37	-2.37	1.37	1.51	0.51	0.78	0.26
	alr2_2fzd_alr2_1iei_pose_03	1.26	-1.7	1.69	2.18	0.86	0.78	0.26
	alr2_2fzd_alr2_1iei_pose_04	1.88	-1.08	2.14	1.77	0.66	0.57	0.34
	alr2_2fzd_alr2_1iei_pose_05	7.06	-0.59	1.96	2.59	0.92	0.64	0.27
2	alr2_2fzd_alr2_1t40_pose_01	2.35	-0.91	2.11	2.23	0.83	0.63	0.31
	alr2_2fzd_alr2_1t40_pose_02	10.5	1.02	2.66	3.95	1.64	0.59	0.31
	alr2_2fzd_alr2_1t40_pose_03	1.05	-2.61	1.24	1.45	0.33	0.81	0.22
	alr2_2fzd_alr2_1t40_pose_04	2.47	-2.25	1.49	1.89	0.98	0.83	0.21
	alr2_2fzd_alr2_1t40_pose_05	5.32	-0.99	2.72	2.52	1.77	0.7	0.28
3	alr2_2fzd_alr2_1z3n_pose_01	0.6	-2.47	1.4	1.24	0.38	0.74	0.26
	alr2_2fzd_alr2_1z3n_pose_02	0.53	-1.97	1.54	1.61	0.53	0.72	0.26
	alr2_2fzd_alr2_1z3n_pose_03	2.33	-2.07	1.44	1.54	0.39	0.72	0.25
	alr2_2fzd_alr2_1z3n_pose_04	0.69	-2.61	1.27	1.16	0.3	0.75	0.23
	alr2_2fzd_alr2_1z3n_pose_05	0.68	-2.36	1.45	1.18	0.31	0.71	0.27
4	alr2_2fzd_alr2_2hvn_pose_01	0.4	-2.63	1.35	1.22	0.3	0.77	0.26
	alr2_2fzd_alr2_2hvn_pose_02	5.77	-2.3	1.68	1.54	0.65	0.77	0.26
	alr2_2fzd_alr2_2hvn_pose_03	1.36	-2.47	1.45	-1.43	-0.34	0.78	0.27
	alr2_2fzd_alr2_2hvn_pose_04	9.5	-0.23	2.79	3.02	1.75	0.65	0.33
	alr2_2fzd_alr2_2hvn_pose_05	9.87	-0.01	1.57	3.67	0.49	0.74	0.28
5	alr2_2fzd_alr2_2ikg_pose_01	5.81	1.85	2.25	5.1	1.58	0.65	0.32
	alr2_2fzd_alr2_2ikg_pose_02	6.03	2.14	1.66	5.24	1.16	0.62	0.32
	alr2_2fzd_alr2_2ikg_pose_03	0.79	-2.49	1.41	1.19	0.37	0.74	0.26
	alr2_2fzd_alr2_2ikg_pose_04	5.22	1.48	2.56	4.42	1.82	0.59	0.31
	alr2_2fzd_alr2_2ikg_pose_05	4.75	-0.31	1.89	3.35	0.92	0.73	0.3
6	cdk2_1pxj_cdk2_1jvp_pose_01	0.61	-0.9	1.83	2.34	1.13	0.65	0.33
	cdk2_1pxj_cdk2_1jvp_pose_02	6.32	0.31	2.71	2.47	1.05	0.43	0.42
	cdk2_1pxj_cdk2_1jvp_pose_03	0.75	-1.57	1.73	2.06	0.98	0.73	0.31
	cdk2_1pxj_cdk2_1jvp_pose_04	6.42	0.6	3.01	2.75	1.68	0.43	0.37
	cdk2_1pxj_cdk2_1jvp_pose_05	5.28	6.8	6.61	7.47	6.14	0.13	2.29

System ID	pose_name	pose_rmsd	CompScore	CompScoreSD	PoseScore	PoseScoreSD	ContactScore	ContactScoreSD
7	cdk2_1pxj_cdk2_1ke8_pose_01	2.13	-1.51	1.42	2.2	0.89	0.74	0.25
	cdk2_1pxj_cdk2_1ke8_pose_02	10.34	-0.9	3.14	2.22	1.86	0.62	0.32
	cdk2_1pxj_cdk2_1ke8_pose_03	1.92	-1.15	1.95	2.49	1.13	0.73	0.26
	cdk2_1pxj_cdk2_1ke8_pose_04	1.64	-1.67	1.48	2.04	0.75	0.74	0.27
	cdk2_1pxj_cdk2_1ke8_pose_05	1.32	-1.8	1.59	2.05	0.79	0.77	0.27
8	cdk2_1pxj_cdk2_1pxo_pose_01	0.96	-2.37	1.48	1.59	0.71	0.79	0.25
	cdk2_1pxj_cdk2_1pxo_pose_02	1.04	-2.3	1.47	1.55	0.58	0.77	0.25
	cdk2_1pxj_cdk2_1pxo_pose_03	2.83	-2.06	1.74	1.63	0.69	0.74	0.26
	cdk2_1pxj_cdk2_1pxo_pose_04	1.63	-2.44	1.63	1.61	0.89	0.81	0.22
	cdk2_1pxj_cdk2_1pxo_pose_05	2.48	0.83	4.24	3.37	2.82	0.51	0.39
9	cdk2_1pxj_cdk2_2a0c_pose_01	1.49	-0.24	1.76	2.75	0.4	0.6	0.32
	cdk2_1pxj_cdk2_2a0c_pose_02	2.21	-0.52	2.12	2.6	0.69	0.62	0.36
	cdk2_1pxj_cdk2_2a0c_pose_03	0.9	-0.63	1.94	2.68	0.53	0.66	0.34
	cdk2_1pxj_cdk2_2a0c_pose_04	0.83	-0.38	2.1	2.67	0.61	0.61	0.35
	cdk2_1pxj_cdk2_2a0c_pose_05	1.61	-0.48	1.86	2.87	0.59	0.67	0.34
10	cdk2_1pxj_cdk2_2btr_pose_01	0.98	-1.44	1.78	2.17	0.5	0.72	0.34
	cdk2_1pxj_cdk2_2btr_pose_02	0.94	-1.58	1.68	1.93	0.56	0.7	0.32
	cdk2_1pxj_cdk2_2btr_pose_03	0.5	-1.93	2.01	1.83	0.7	0.75	0.35
	cdk2_1pxj_cdk2_2btr_pose_04	1.44	-1.4	1.71	2.01	0.6	0.68	0.31
	cdk2_1pxj_cdk2_2btr_pose_05	7.88	4.22	3.31	5.73	1.82	0.3	0.37
11	cdk2_1pxj_cdk2_2bts_pose_01	1.1	-1.17	1.64	2.04	0.53	0.64	0.32
	cdk2_1pxj_cdk2_2bts_pose_02	1.31	-1.37	1.62	2.32	0.41	0.74	0.3
	cdk2_1pxj_cdk2_2bts_pose_03	8.15	0.01	3.0	2.89	1.21	0.58	0.39
	cdk2_1pxj_cdk2_2bts_pose_04	7.6	4.18	3.59	5.17	2.36	0.2	0.33
	cdk2_1pxj_cdk2_2bts_pose_05	2.13	3.37	6.34	5.29	5.19	0.39	0.42
12	cdk2_1pxj_cdk2_2c6m_pose_01	7.77	2.88	3.51	4.41	2.23	0.31	0.34
	cdk2_1pxj_cdk2_2c6m_pose_02	8.72	5.11	3.52	5.95	2.9	0.17	0.24
	cdk2_1pxj_cdk2_2c6m_pose_03	1.04	-1.64	1.5	2.12	0.44	0.75	0.28
	cdk2_1pxj_cdk2_2c6m_pose_04	8.69	8.49	5.59	8.94	5.11	0.09	0.25
	cdk2_1pxj_cdk2_2c6m_pose_05	4.93	8.52	6.6	9.13	5.87	0.12	0.28
13	cdk2_1wcc_cdk2_1oit_pose_01	1.9	0.28	2.2	3.29	0.93	0.6	0.38
	cdk2_1wcc_cdk2_1oit_pose_02	6.71	1.24	3.84	3.41	2.74	0.43	0.31
	cdk2_1wcc_cdk2_1oit_pose_03	1.16	0.56	2.1	2.92	1.02	0.47	0.31
	cdk2_1wcc_cdk2_1oit_pose_04	6.86	0.39	2.81	2.7	1.47	0.46	0.34
	cdk2_1wcc_cdk2_1oit_pose_05	6.57	3.4	3.75	4.89	2.76	0.3	0.33

System ID	pose_name	pose_rmsd	CompScore	CompScoreSD	PoseScore	PoseScoreSD	ContactScore	ContactScoreSD
14	cdk2_1wcc_cdk2_2b52_pose_01	8.08	1.32	2.73	3.75	1.94	0.49	0.3
	cdk2_1wcc_cdk2_2b52_pose_02	6.24	-1.61	1.87	2.1	0.82	0.74	0.31
	cdk2_1wcc_cdk2_2b52_pose_03	7.96	5.04	7.21	6.81	5.95	0.35	0.41
	cdk2_1wcc_cdk2_2b52_pose_04	1.39	-0.4	2.08	2.93	0.74	0.67	0.33
	cdk2_1wcc_cdk2_2b52_pose_05	1.85	0.83	2.12	3.57	0.65	0.55	0.36
15	cdk2_1wcc_cdk2_2bhe_pose_01	0.88	-2.89	1.32	1.31	0.39	0.84	0.25
	cdk2_1wcc_cdk2_2bhe_pose_02	0.36	-2.12	1.47	1.47	0.5	0.72	0.29
	cdk2_1wcc_cdk2_2bhe_pose_03	6.28	1.01	6.51	3.7	4.96	0.54	0.43
	cdk2_1wcc_cdk2_2bhe_pose_04	4.49	-0.18	3.42	2.54	2.15	0.54	0.41
	cdk2_1wcc_cdk2_2bhe_pose_05	1.18	-2.41	1.37	1.4	0.36	0.76	0.27
16	cdk2_1wcc_cdk2_2bts_pose_01	2.77	-1.59	1.76	2.07	0.52	0.73	0.33
	cdk2_1wcc_cdk2_2bts_pose_02	2.54	-1.36	1.64	2.36	0.63	0.74	0.3
	cdk2_1wcc_cdk2_2bts_pose_03	0.54	-1.28	1.83	1.84	0.65	0.62	0.29
	cdk2_1wcc_cdk2_2bts_pose_04	0.69	-1.0	2.25	2.42	1.13	0.68	0.28
	cdk2_1wcc_cdk2_2bts_pose_05	1.07	-1.73	1.74	2.02	0.58	0.75	0.29
17	cdk2_1wcc_cdk2_2c6k_pose_01	0.75	-1.49	1.47	2.3	0.56	0.76	0.27
	cdk2_1wcc_cdk2_2c6k_pose_02	0.85	-1.4	1.6	2.1	0.61	0.7	0.3
	cdk2_1wcc_cdk2_2c6k_pose_03	1.63	-1.49	1.78	2.45	0.76	0.79	0.3
	cdk2_1wcc_cdk2_2c6k_pose_04	1.2	-0.98	2.02	2.61	0.95	0.72	0.3
	cdk2_1wcc_cdk2_2c6k_pose_05	6.99	0.32	1.94	2.78	0.78	0.49	0.32
18	chk1_2c3j_chk1_1nvs_pose_01	1.22	-1.77	1.9	1.74	0.8	0.7	0.31
	chk1_2c3j_chk1_1nvs_pose_02	1.01	-2.04	1.76	1.41	0.56	0.69	0.32
	chk1_2c3j_chk1_1nvs_pose_03	4.66	7.28	5.02	7.56	4.81	0.06	0.16
	chk1_2c3j_chk1_1nvs_pose_04	4.82	4.84	3.83	5.63	2.69	0.16	0.32
	chk1_2c3j_chk1_1nvs_pose_05	5.04	2.6	3.88	3.98	2.29	0.28	0.4
19	chk1_2c3j_chk1_2cgu_pose_01	1.68	-1.66	1.9	1.76	0.99	0.68	0.26
	chk1_2c3j_chk1_2cgu_pose_02	2.14	-1.12	2.07	2.22	1.17	0.67	0.3
	chk1_2c3j_chk1_2cgu_pose_03	3.03	-0.76	2.01	2.82	1.15	0.72	0.3
	chk1_2c3j_chk1_2cgu_pose_04	2.25	-1.39	2.6	2.17	1.75	0.71	0.29
	chk1_2c3j_chk1_2cgu_pose_05	3.98	0.43	2.14	2.77	1.12	0.47	0.36
20	chk1_2e9o_chk1_2cgu_pose_01	1.54	-1.55	1.78	1.97	0.86	0.7	0.27
	chk1_2e9o_chk1_2cgu_pose_02	2.69	-1.5	1.62	1.84	0.58	0.67	0.29
	chk1_2e9o_chk1_2cgu_pose_03	2.23	-1.28	1.85	2.05	0.88	0.66	0.31
	chk1_2e9o_chk1_2cgu_pose_04	1.98	-1.16	1.74	1.95	0.73	0.62	0.3
	chk1_2e9o_chk1_2cgu_pose_05	2.35	-1.48	1.96	1.99	0.84	0.69	0.3

System ID	pose_name	pose_rmsd	CompScore	CompScoreSD	PoseScore	PoseScoreSD	ContactScore	ContactScoreSD
21	chk1_2e9o_chk1_2cgx_pose_01	1.93	-0.53	2.29	2.38	0.98	0.58	0.35
	chk1_2e9o_chk1_2cgx_pose_02	2.03	-0.49	2.13	2.54	0.89	0.61	0.38
	chk1_2e9o_chk1_2cgx_pose_03	1.66	-0.48	2.83	2.69	1.39	0.63	0.38
	chk1_2e9o_chk1_2cgx_pose_04	2.71	0.72	1.98	3.42	0.85	0.54	0.33
	chk1_2e9o_chk1_2cgx_pose_05	2.92	-1.01	2.28	2.45	1.14	0.69	0.33
22	dpp4_1n1m_dpp4_1x70_pose_01	8.59	1.51	1.88	3.52	0.93	0.4	0.3
	dpp4_1n1m_dpp4_1x70_pose_02	3.19	1.89	2.22	5.04	1.81	0.63	0.28
	dpp4_1n1m_dpp4_1x70_pose_03	6.64	0.47	2.6	3.8	1.69	0.66	0.33
	dpp4_1n1m_dpp4_1x70_pose_04	0.46	-0.02	2.76	2.75	1.63	0.55	0.31
	dpp4_1n1m_dpp4_1x70_pose_05	3.54	2.57	2.68	4.54	2.16	0.39	0.29
23	dpp4_1rwq_dpp4_1x70_pose_01	2.84	1.45	2.49	4.03	1.39	0.52	0.35
	dpp4_1rwq_dpp4_1x70_pose_02	2.52	-0.74	1.56	2.68	0.91	0.68	0.22
	dpp4_1rwq_dpp4_1x70_pose_03	4.61	-0.65	1.42	2.89	0.55	0.71	0.26
	dpp4_1rwq_dpp4_1x70_pose_04	1.9	1.28	3.02	3.73	2.14	0.49	0.28
	dpp4_1rwq_dpp4_1x70_pose_05	3.66	1.56	2.29	4.6	1.32	0.61	0.31
24	fxa_1g2m_fxa_1lpk_pose_01	4.38	0.66	2.15	3.83	1.52	0.63	0.25
	fxa_1g2m_fxa_1lpk_pose_02	7.96	-0.84	3.23	2.73	1.95	0.71	0.3
	fxa_1g2m_fxa_1lpk_pose_03	1.17	-2.23	1.64	1.82	0.76	0.81	0.22
	fxa_1g2m_fxa_1lpk_pose_04	3.09	-1.82	1.3	1.96	0.47	0.76	0.23
	fxa_1g2m_fxa_1lpk_pose_05	7.96	1.63	1.69	5.07	1.17	0.69	0.23
25	fxa_1nfx_fxa_1lpk_pose_01	1.98	-1.39	1.41	2.27	0.72	0.73	0.22
	fxa_1nfx_fxa_1lpk_pose_02	0.79	-2.29	1.51	1.63	0.71	0.79	0.23
	fxa_1nfx_fxa_1lpk_pose_03	7.8	-1.01	2.83	2.84	2.15	0.77	0.22
	fxa_1nfx_fxa_1lpk_pose_04	9.04	0.9	2.33	4.49	1.52	0.72	0.27
	fxa_1nfx_fxa_1lpk_pose_05	7.64	1.2	2.3	4.56	1.79	0.67	0.25
26	fxa_1nfx_fxa_1lpz_pose_01	0.54	-1.65	1.74	2.48	1.22	0.83	0.2
	fxa_1nfx_fxa_1lpz_pose_02	6.31	1.31	2.4	4.68	1.9	0.67	0.26
	fxa_1nfx_fxa_1lpz_pose_03	0.82	-3.01	1.22	1.09	0.3	0.82	0.22
	fxa_1nfx_fxa_1lpz_pose_04	9.7	2.39	2.32	3.75	1.05	0.27	0.38
	fxa_1nfx_fxa_1lpz_pose_05	6.58	2.57	3.98	4.56	2.87	0.4	0.29
27	fxa_1nfx_fxa_1lqd_pose_01	1.58	-2.45	1.31	1.6	0.6	0.81	0.22
	fxa_1nfx_fxa_1lqd_pose_02	1.14	-2.84	1.07	1.61	0.54	0.89	0.19
	fxa_1nfx_fxa_1lqd_pose_03	3.75	1.58	1.88	5.37	1.3	0.76	0.24
	fxa_1nfx_fxa_1lqd_pose_04	4.54	1.04	2.55	4.39	1.6	0.67	0.29
	fxa_1nfx_fxa_1lqd_pose_05	6.17	-1.36	1.58	2.56	0.71	0.79	0.24

System ID	pose_name	pose_rmsd	CompScore	CompScoreSD	PoseScore	PoseScoreSD	ContactScore	ContactScoreSD
28	fxa_1xka_fxa_1lqd_pose_01	0.84	-1.06	2.27	2.79	1.94	0.77	0.22
	fxa_1xka_fxa_1lqd_pose_02	4.42	1.08	2.35	4.39	1.65	0.66	0.28
	fxa_1xka_fxa_1lqd_pose_03	6.74	-0.46	1.6	3.4	1.15	0.77	0.24
	fxa_1xka_fxa_1lqd_pose_04	7.51	4.61	4.77	6.21	3.43	0.32	0.35
	fxa_1xka_fxa_1lqd_pose_05	6.74	-0.92	1.55	2.86	1.0	0.76	0.24
29	pka_2c1a_pka_1bx6_pose_01	1.56	-1.61	1.34	1.8	0.46	0.68	0.23
	pka_2c1a_pka_1bx6_pose_02	6.37	-1.59	1.75	2.03	0.64	0.72	0.26
	pka_2c1a_pka_1bx6_pose_03	6.0	-1.33	1.64	2.13	0.59	0.69	0.26
	pka_2c1a_pka_1bx6_pose_04	5.81	-0.28	1.34	2.41	0.57	0.54	0.22
	pka_2c1a_pka_1bx6_pose_05	2.64	-1.33	1.18	1.93	0.3	0.65	0.22
30	pka_2erz_pka_1cdk_pose_01	1.02	20.58	14.44	20.87	14.11	0.06	0.18
	pka_2erz_pka_1cdk_pose_02	1.89	24.09	14.12	24.18	14.06	0.02	0.11
	pka_2erz_pka_1cdk_pose_03	2.69	7.1	8.18	7.89	7.7	0.16	0.22
	pka_2erz_pka_1cdk_pose_04	2.31	5.76	6.59	6.9	5.75	0.23	0.29
	pka_2erz_pka_1cdk_pose_05	1.63	12.61	9.4	12.79	9.2	0.04	0.17
31	pka_2erz_pka_1svg_pose_01	1.53	-2.69	1.11	1.41	0.67	0.82	0.19
	pka_2erz_pka_1svg_pose_02	3.36	0.28	2.17	3.22	1.25	0.59	0.26
	pka_2erz_pka_1svg_pose_03	9.86	0.46	1.8	2.84	0.96	0.48	0.27
	pka_2erz_pka_1svg_pose_04	10.3	-1.48	1.71	1.98	0.8	0.69	0.26
	pka_2erz_pka_1svg_pose_05	4.42	2.59	2.48	4.29	1.74	0.34	0.3
32	ppar_2prg_ppar_1zgy_pose_01	7.02	1.45	2.29	2.98	1.11	0.31	0.34
	ppar_2prg_ppar_1zgy_pose_02	1.04	-0.32	2.69	2.25	1.04	0.51	0.41
	ppar_2prg_ppar_1zgy_pose_03	6.73	3.58	1.49	4.09	0.97	0.1	0.22
	ppar_2prg_ppar_1zgy_pose_04	5.93	2.24	2.83	3.67	1.42	0.29	0.38
	ppar_2prg_ppar_1zgy_pose_05	7.08	3.19	3.04	4.84	1.82	0.33	0.43
33	ppar_2prg_ppar_2ath_pose_01	9.53	0.62	2.49	2.97	0.89	0.47	0.41
	ppar_2prg_ppar_2ath_pose_02	9.4	2.56	2.79	4.49	1.34	0.39	0.37
	ppar_2prg_ppar_2ath_pose_03	4.83	0.86	2.38	3.46	1.27	0.52	0.41
	ppar_2prg_ppar_2ath_pose_04	3.14	0.94	1.85	2.68	0.53	0.35	0.35
	ppar_2prg_ppar_2ath_pose_05	1.71	-1.22	1.75	2.18	0.55	0.68	0.32
34	ppar_2prg_ppar_2gtk_pose_01	0.65	-0.64	2.66	2.06	1.05	0.54	0.4
	ppar_2prg_ppar_2gtk_pose_02	2.27	-0.27	2.22	2.65	0.91	0.58	0.34
	ppar_2prg_ppar_2gtk_pose_03	2.12	-0.29	2.01	2.8	0.61	0.62	0.34
	ppar_2prg_ppar_2gtk_pose_04	2.07	0.13	2.08	3.14	0.75	0.6	0.37
	ppar_2prg_ppar_2gtk_pose_05	0.96	-1.11	2.05	2.18	0.72	0.66	0.33

System ID	pose_name	pose_rmsd	CompScore	CompScoreSD	PoseScore	PoseScoreSD	ContactScore	ContactScoreSD
35	throm_1ae8_throm_1gj4_pose_01	2.11	2.46	2.03	4.76	1.2	0.46	0.27
	throm_1ae8_throm_1gj4_pose_02	1.49	-3.07	1.2	1.17	0.42	0.85	0.2
	throm_1ae8_throm_1gj4_pose_03	1.61	-3.43	0.98	0.92	0.39	0.87	0.17
	throm_1ae8_throm_1gj4_pose_04	3.39	0.46	2.99	3.27	1.69	0.56	0.36
	throm_1ae8_throm_1gj4_pose_05	6.69	0.81	2.89	3.94	1.84	0.62	0.32
36	throm_1ae8_throm_1gj5_pose_01	6.14	-2.0	1.94	2.17	1.19	0.83	0.22
	throm_1ae8_throm_1gj5_pose_02	2.23	2.12	3.37	4.61	1.72	0.5	0.4
	throm_1ae8_throm_1gj5_pose_03	2.39	3.74	3.92	5.97	2.48	0.45	0.37
	throm_1ae8_throm_1gj5_pose_04	0.78	-2.26	1.82	1.73	0.98	0.8	0.23
	throm_1ae8_throm_1gj5_pose_05	6.17	0.22	2.63	3.33	1.56	0.62	0.29
37	throm_1ae8_throm_1o2g_pose_01	0.69	-2.19	1.36	1.7	0.79	0.78	0.22
	throm_1ae8_throm_1o2g_pose_02	1.35	-3.1	1.11	1.04	0.37	0.83	0.10
	throm_1ae8_throm_1o2g_pose_03	3.55	-0.45	1.58	3.39	1.03	0.77	0.25
	throm_1ae8_throm_1o2g_pose_04	5.59	-0.29	2.76	3.29	1.83	0.72	0.27
	throm_1ae8_throm_1o2g_pose_05	1.67	-2.98	1.29	1.11	0.47	0.82	0.22
38	throm_1ae8_throm_1o5g_pose_01	0.99	-2.36	1.77	1.83	1.14	0.84	0.21
	throm_1ae8_throm_1o5g_pose_02	1.09	-1.71	2.36	2.09	1.28	0.76	0.28
	throm_1ae8_throm_1o5g_pose_03	7.56	2.21	2.51	5.33	1.65	0.62	0.28
	throm_1ae8_throm_1o5g_pose_04	7.32	1.12	3.39	4.35	2.18	0.64	0.3
	throm_1ae8_throm_1o5g_pose_05	2.12	-2.33	1.34	2.11	0.77	0.89	0.19
39	throm_1ae8_throm_1riw_pose_01	1.54	-0.68	1.86	2.82	1.01	0.7	0.26
	throm_1ae8_throm_1riw_pose_02	2.41	-1.81	1.54	2.37	0.94	0.83	0.2
	throm_1ae8_throm_1riw_pose_03	8.96	1.75	3.84	3.98	2.22	0.45	0.42
	throm_1ae8_throm_1riw_pose_04	9.3	1.16	2.5	3.44	1.0	0.46	0.36
	throm_1ae8_throm_1riw_pose_05	6.67	0.09	1.85	3.81	1.15	0.75	0.25
40	throm_1k21_throm_1riw_pose_01	1.48	-0.68	1.38	3.01	0.76	0.74	0.23
	throm_1k21_throm_1riw_pose_02	1.19	-2.16	1.18	2.29	0.94	0.89	0.16
	throm_1k21_throm_1riw_pose_03	2.09	-1.94	1.44	2.31	0.87	0.85	0.19
	throm_1k21_throm_1riw_pose_04	3.02	-1.69	1.53	2.63	1.15	0.86	0.18
	throm_1k21_throm_1riw_pose_05	6.73	0.87	2.28	4.19	1.25	0.67	0.32
41	throm_1nm6_throm_1fpc_pose_01	5.43	3.17	3.53	4.73	2.18	0.31	0.39
	throm_1nm6_throm_1fpc_pose_02	1.28	2.9	2.43	5.79	1.35	0.58	0.34
	throm_1nm6_throm_1fpc_pose_03	1.38	1.46	2.19	4.81	1.71	0.67	0.27
	throm_1nm6_throm_1fpc_pose_04	1.68	0.68	3.36	4.06	2.3	0.68	0.3
	throm_1nm6_throm_1fpc_pose_05	1.22	1.58	2.52	4.52	1.28	0.59	0.34

alr2_1iei_alr2_2fz8

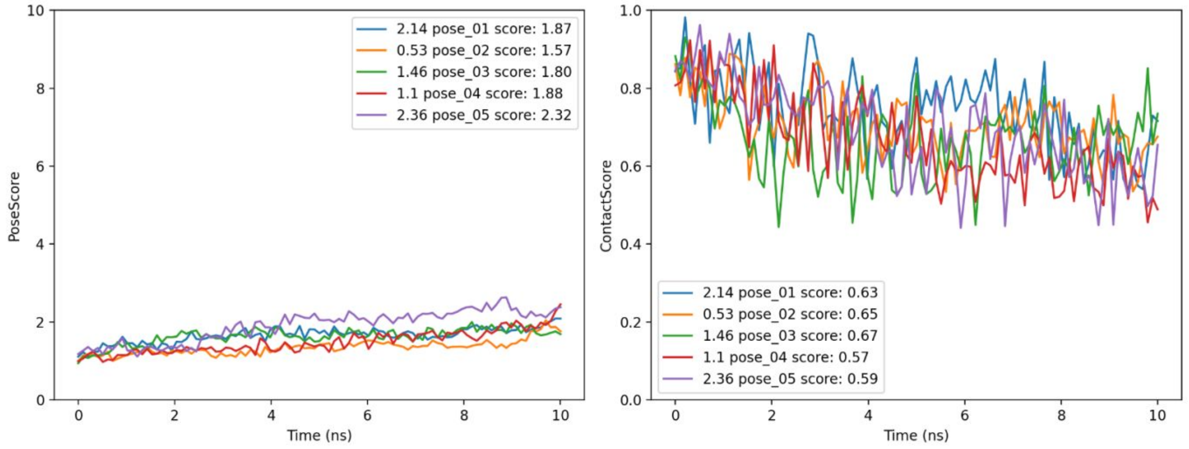

alr2_2fzd_alr2_1iei

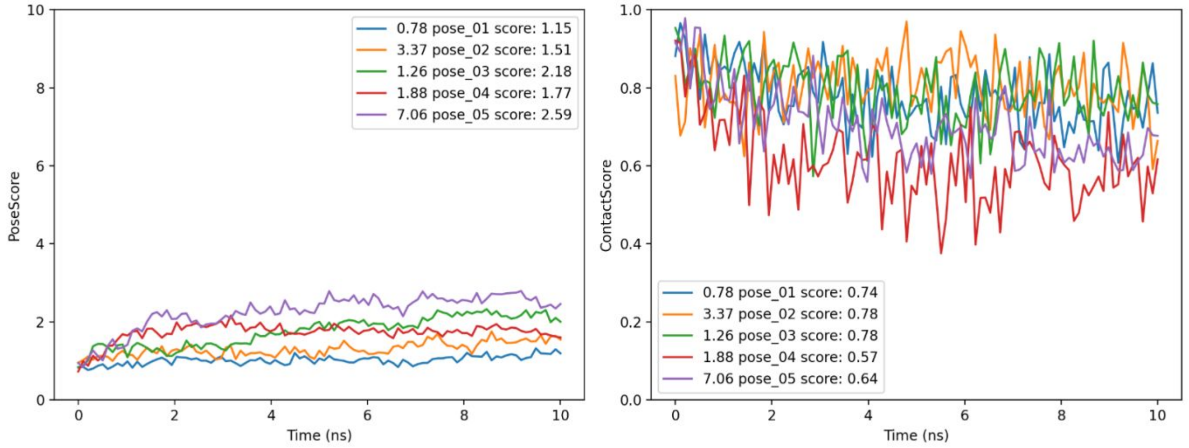

alr2_2fzd_alr2_1t40

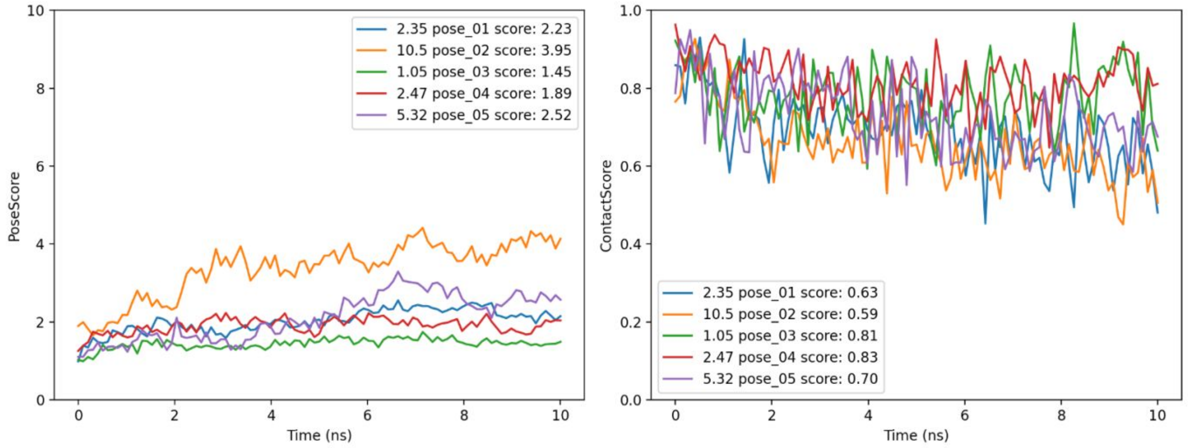

alr2_2fzd_alr2_1z3n

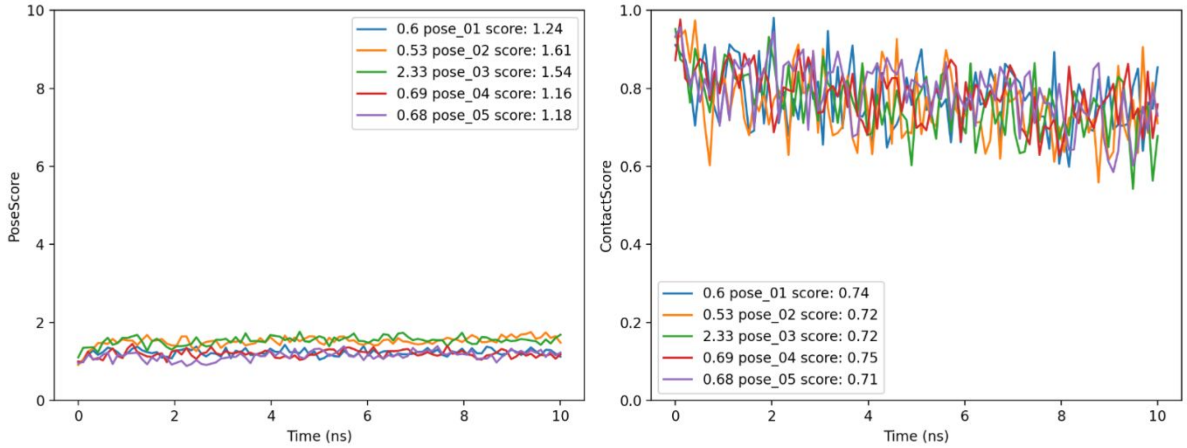

alr2_2fzd_alr2_2hvn

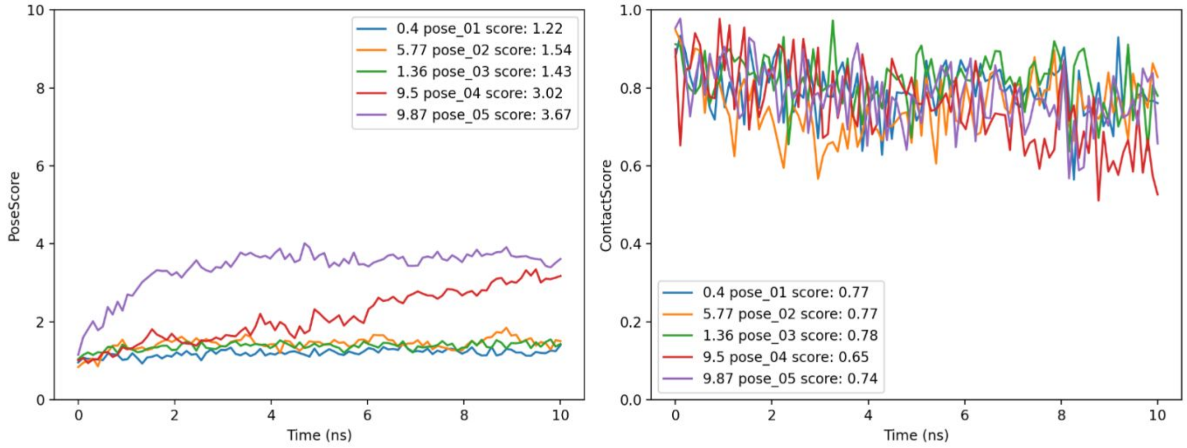

alr2_2fzd_alr2_2ikg

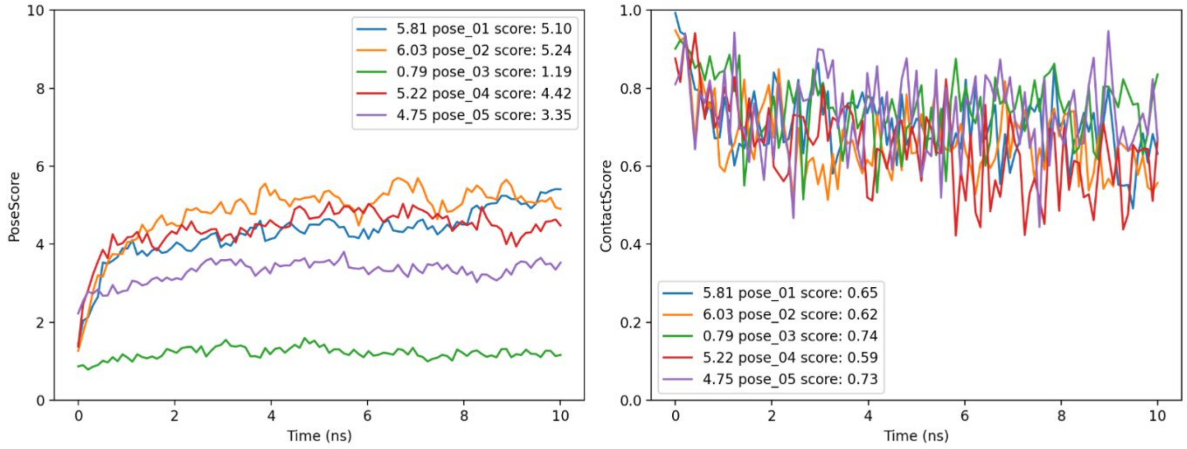

cdk2_1pxj_cdk2_1jvp

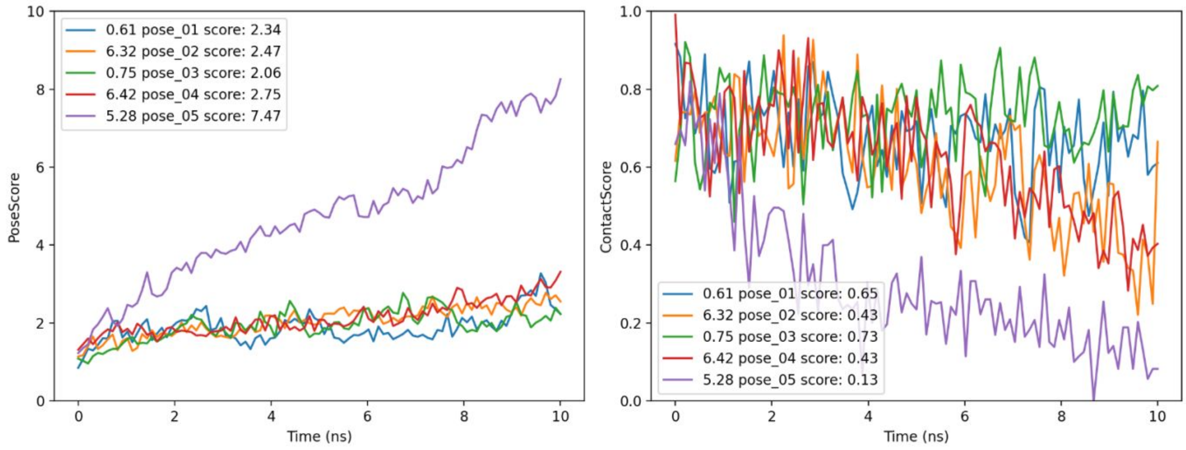

cdk2_1pxj_cdk2_1ke8

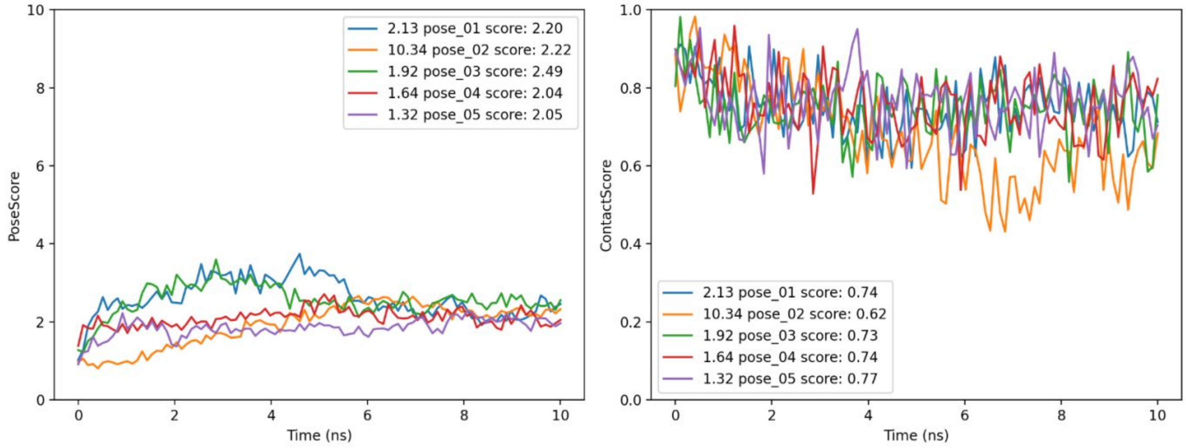

cdk2_1pxj_cdk2_1pxo

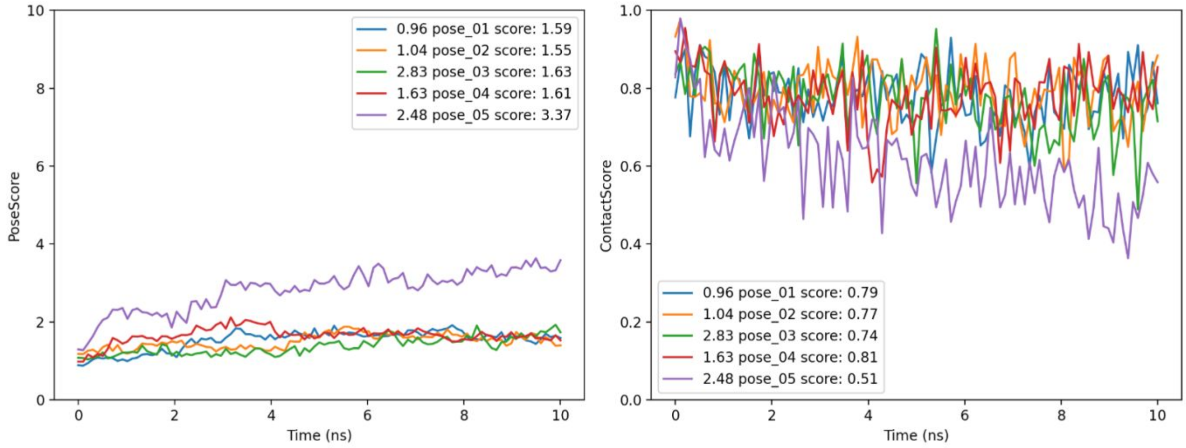

cdk2_1pxj_cdk2_2a0c

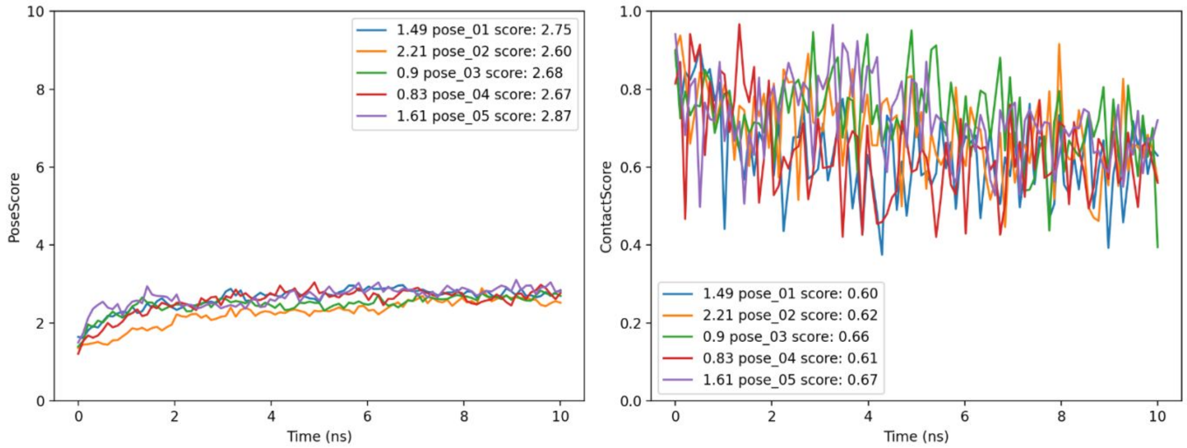

cdk2_1pxj_cdk2_2btr

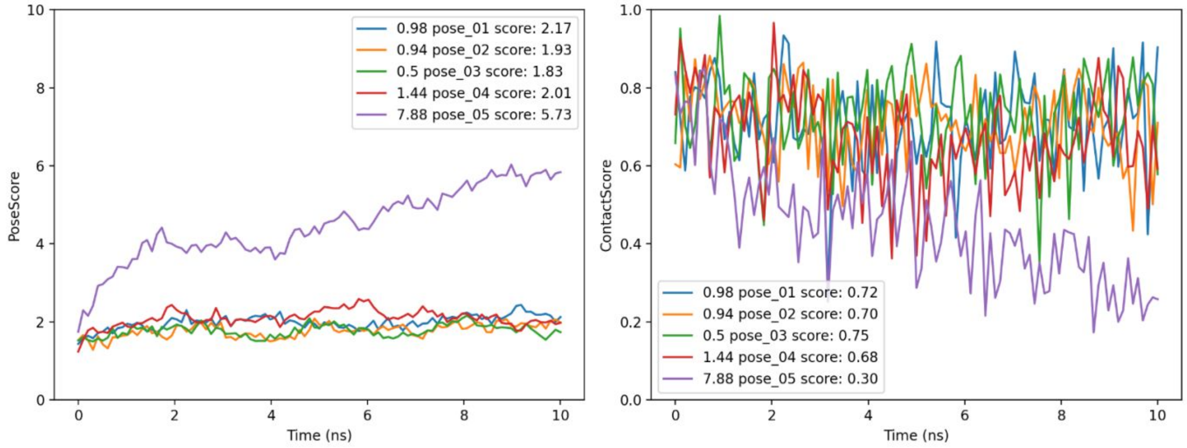

cdk2_1pxj_cdk2_2bts

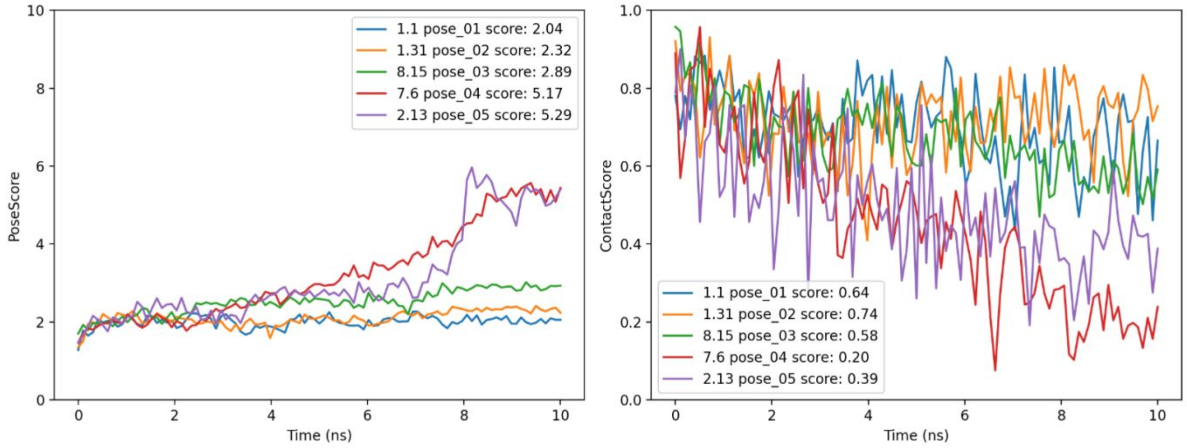

cdk2_1pxj_cdk2_2c6m

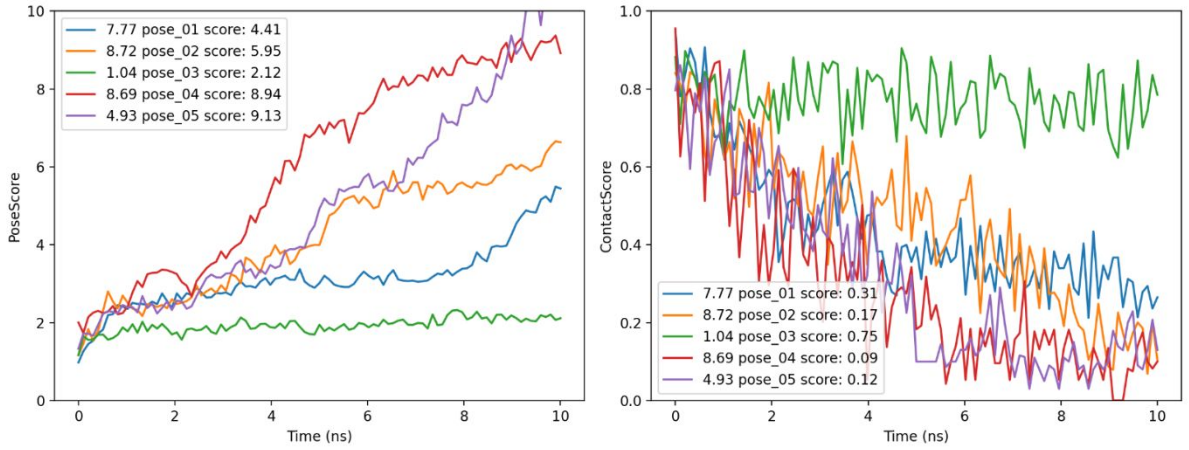

cdk2_1wcc_cdk2_1oit

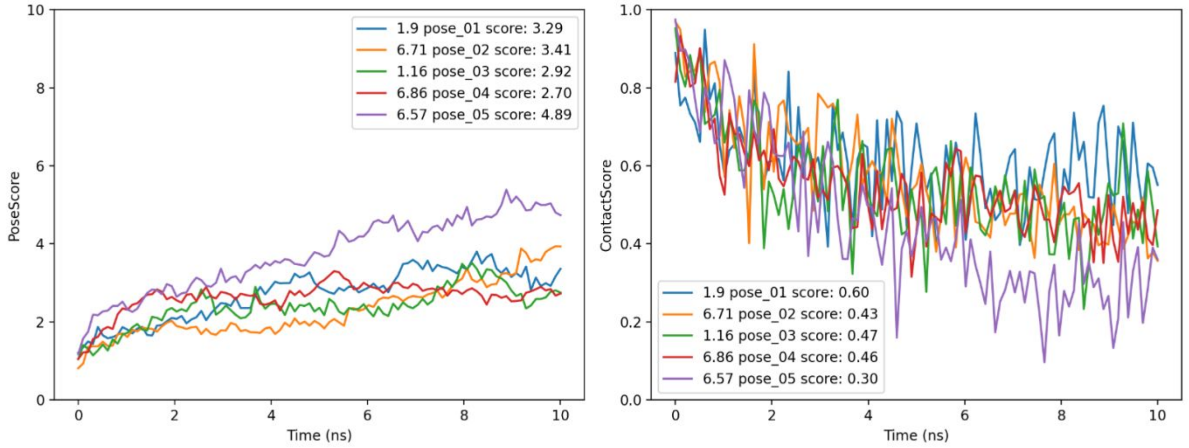

cdk2_1wcc_cdk2_2b52

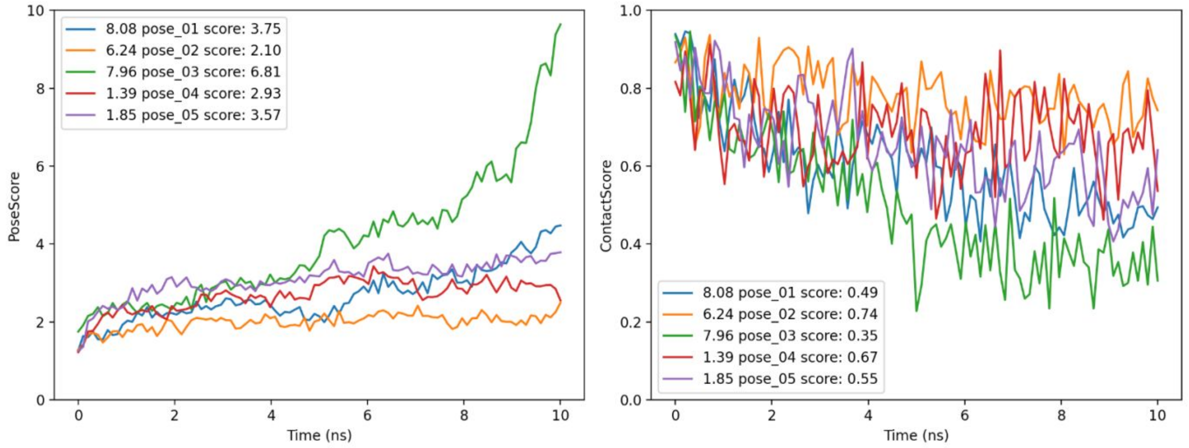

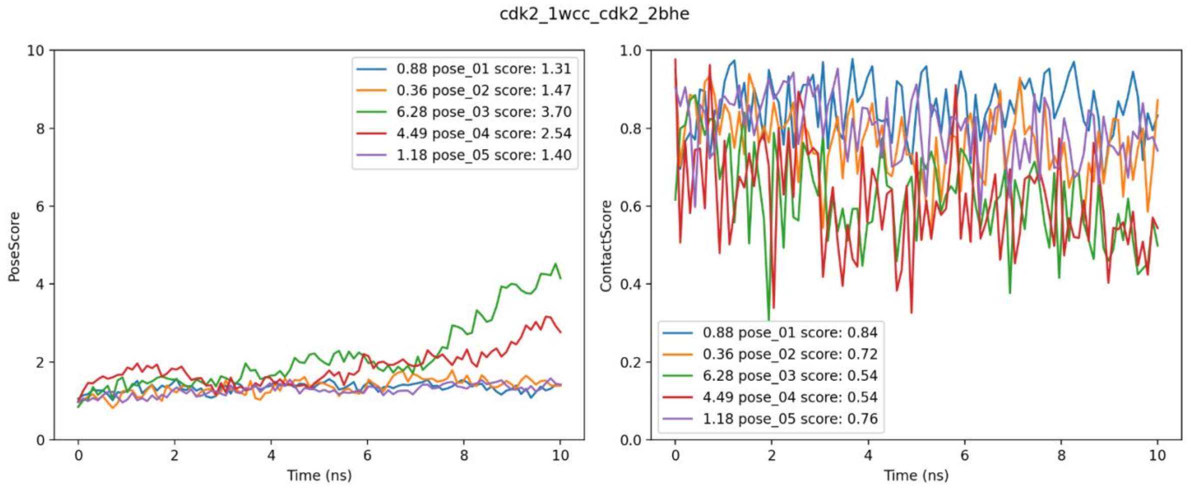
cdk2_1wcc_cdk2_2bhe
PoseScore
Time (ns)
0.88 pose_01 score: 1.31
0.36 pose_02 score: 1.47
6.28 pose_03 score: 3.70
4.49 pose_04 score: 2.54
1.18 pose_05 score: 1.40
ContactScore
Time (ns)
0.88 pose_01 score: 0.84
0.36 pose_02 score: 0.72
6.28 pose_03 score: 0.54
4.49 pose_04 score: 0.54
1.18 pose_05 score: 0.76

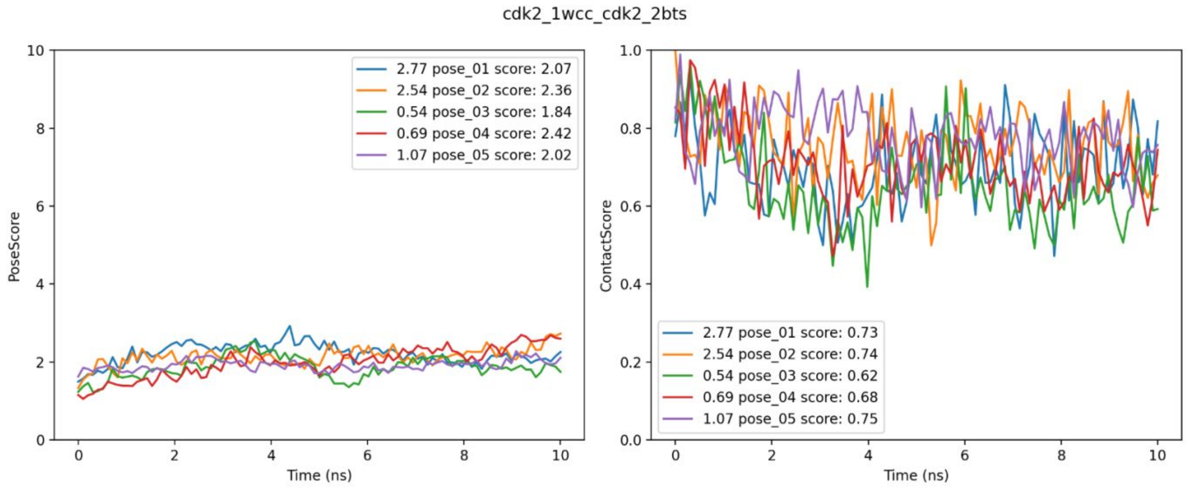
cdk2_1wcc_cdk2_2bts
PoseScore
Time (ns)
2.77 pose_01 score: 2.07
2.54 pose_02 score: 2.36
0.54 pose_03 score: 1.84
0.69 pose_04 score: 2.42
1.07 pose_05 score: 2.02
ContactScore
Time (ns)
2.77 pose_01 score: 0.73
2.54 pose_02 score: 0.74
0.54 pose_03 score: 0.62
0.69 pose_04 score: 0.68
1.07 pose_05 score: 0.75

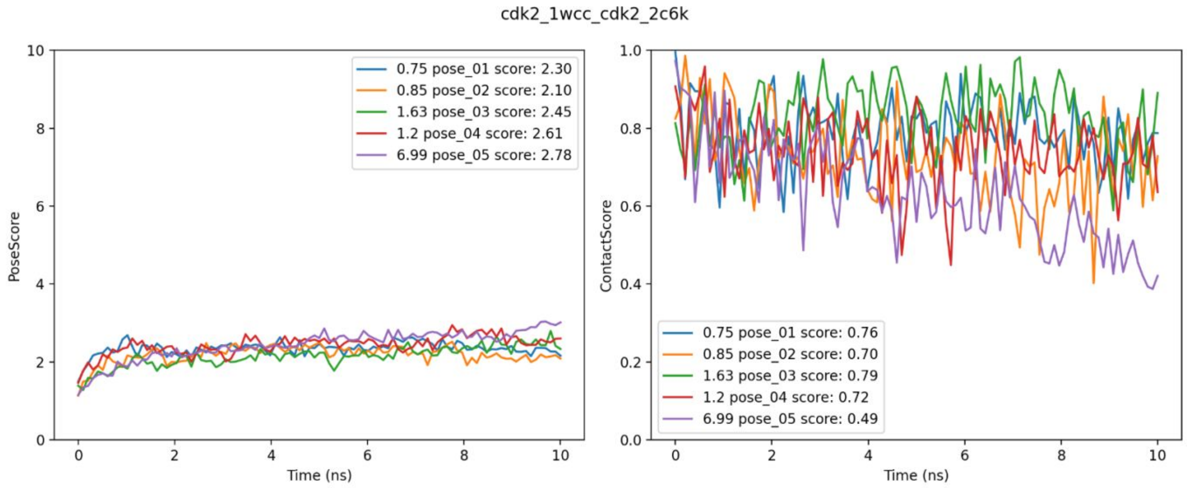
cdk2_1wcc_cdk2_2c6k
PoseScore
Time (ns)
0.75 pose_01 score: 2.30
0.85 pose_02 score: 2.10
1.63 pose_03 score: 2.45
1.2 pose_04 score: 2.61
6.99 pose_05 score: 2.78
ContactScore
Time (ns)
0.75 pose_01 score: 0.76
0.85 pose_02 score: 0.70
1.63 pose_03 score: 0.79
1.2 pose_04 score: 0.72
6.99 pose_05 score: 0.49

chk1_2c3j_chk1_1nvs

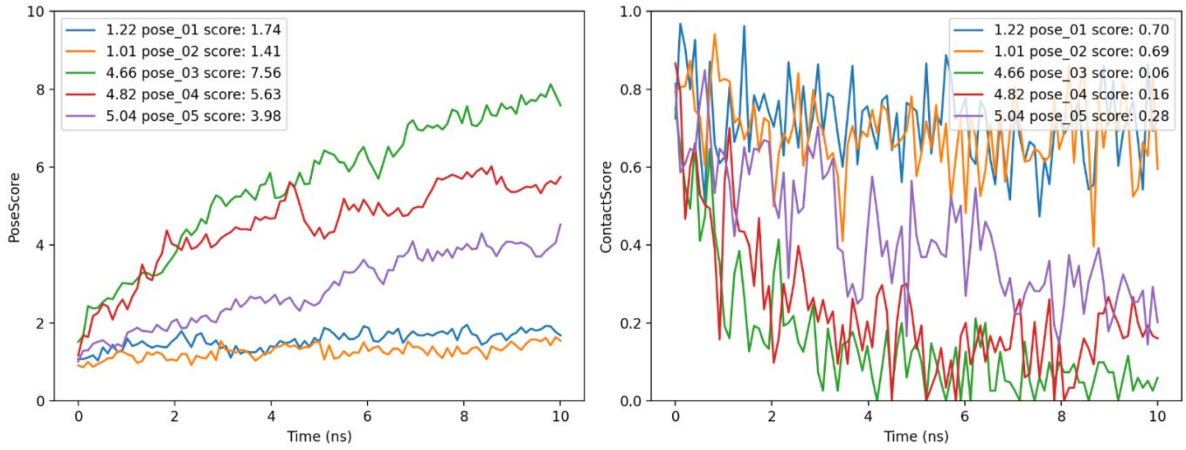

chk1_2c3j_chk1_2cgu

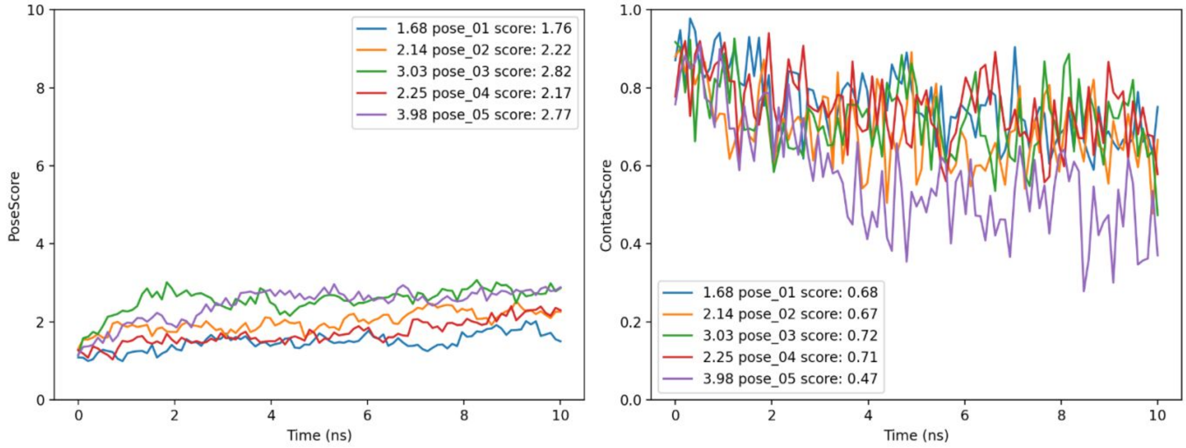

chk1_2e9o_chk1_2cgu

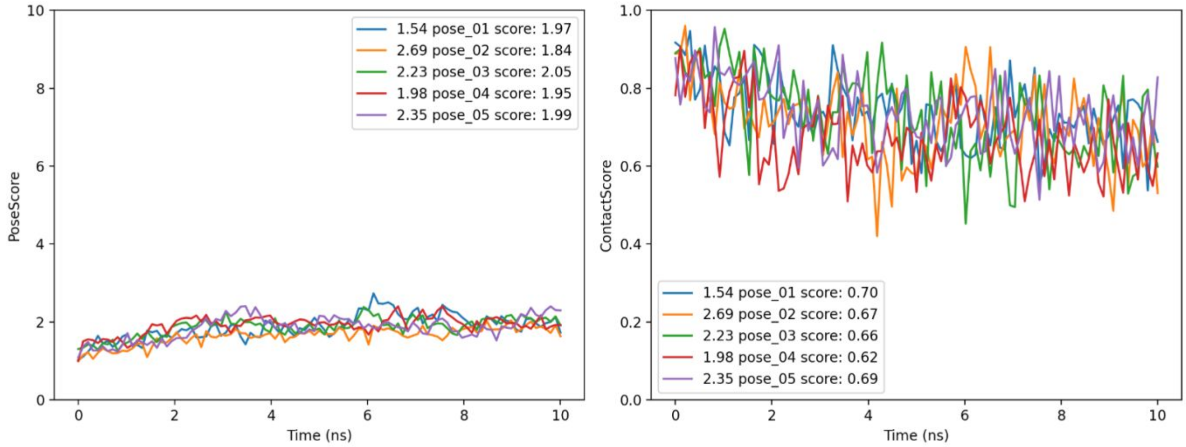

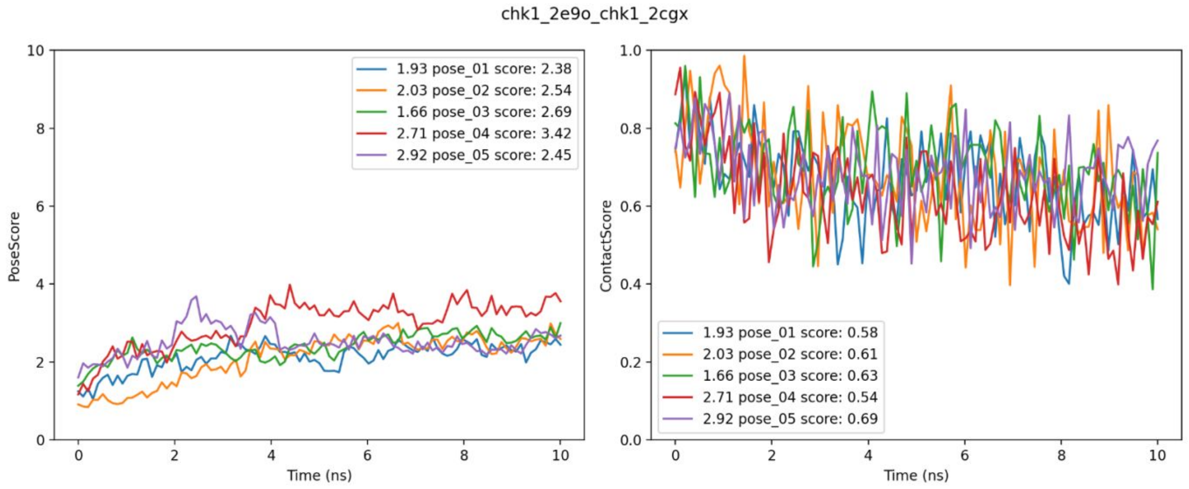
chk1_2e9o_chk1_2cgx
PoseScore
Time (ns)
1.93 pose_01 score: 2.38
2.03 pose_02 score: 2.54
1.66 pose_03 score: 2.69
2.71 pose_04 score: 3.42
2.92 pose_05 score: 2.45
ContactScore
Time (ns)
1.93 pose_01 score: 0.58
2.03 pose_02 score: 0.61
1.66 pose_03 score: 0.63
2.71 pose_04 score: 0.54
2.92 pose_05 score: 0.69

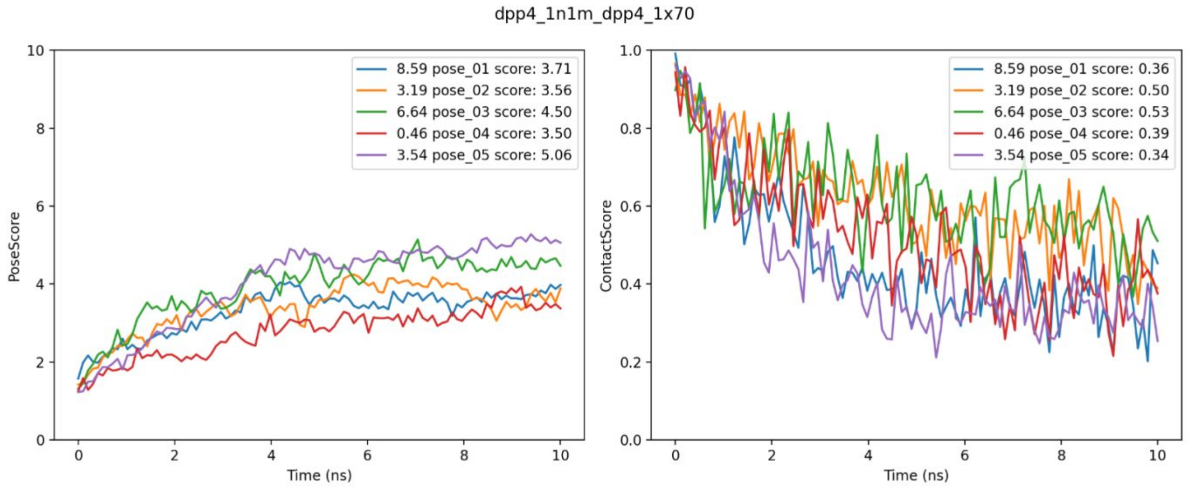
dpp4_1n1m_dpp4_1x70
PoseScore
Time (ns)
8.59 pose_01 score: 3.71
3.19 pose_02 score: 3.56
6.64 pose_03 score: 4.50
0.46 pose_04 score: 3.50
3.54 pose_05 score: 5.06
ContactScore
Time (ns)
8.59 pose_01 score: 0.36
3.19 pose_02 score: 0.50
6.64 pose_03 score: 0.53
0.46 pose_04 score: 0.39
3.54 pose_05 score: 0.34

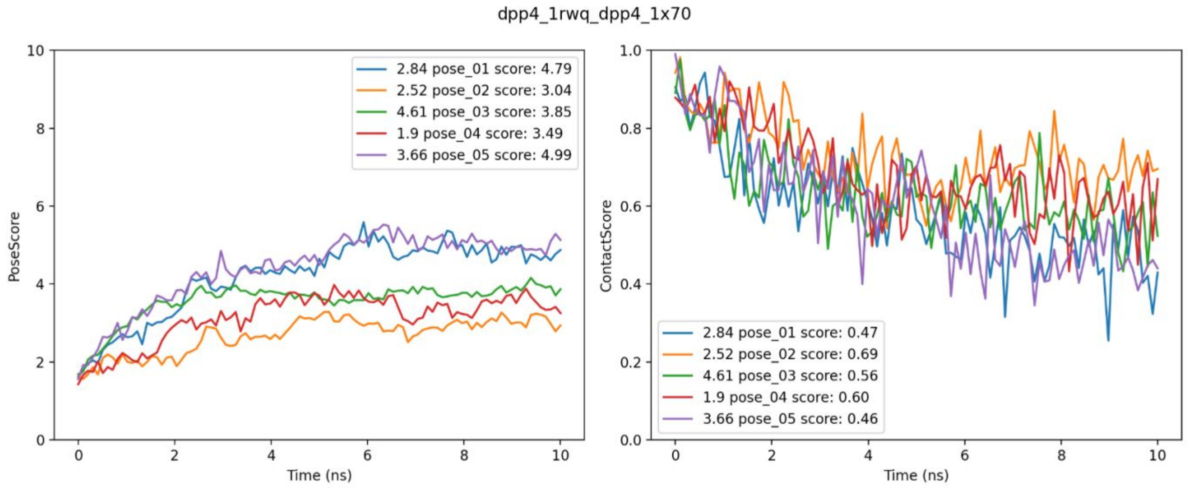
dpp4_1rwq_dpp4_1x70
PoseScore
Time (ns)
2.84 pose_01 score: 4.79
2.52 pose_02 score: 3.04
4.61 pose_03 score: 3.85
1.9 pose_04 score: 3.49
3.66 pose_05 score: 4.99
ContactScore
Time (ns)
2.84 pose_01 score: 0.47
2.52 pose_02 score: 0.69
4.61 pose_03 score: 0.56
1.9 pose_04 score: 0.60
3.66 pose_05 score: 0.46

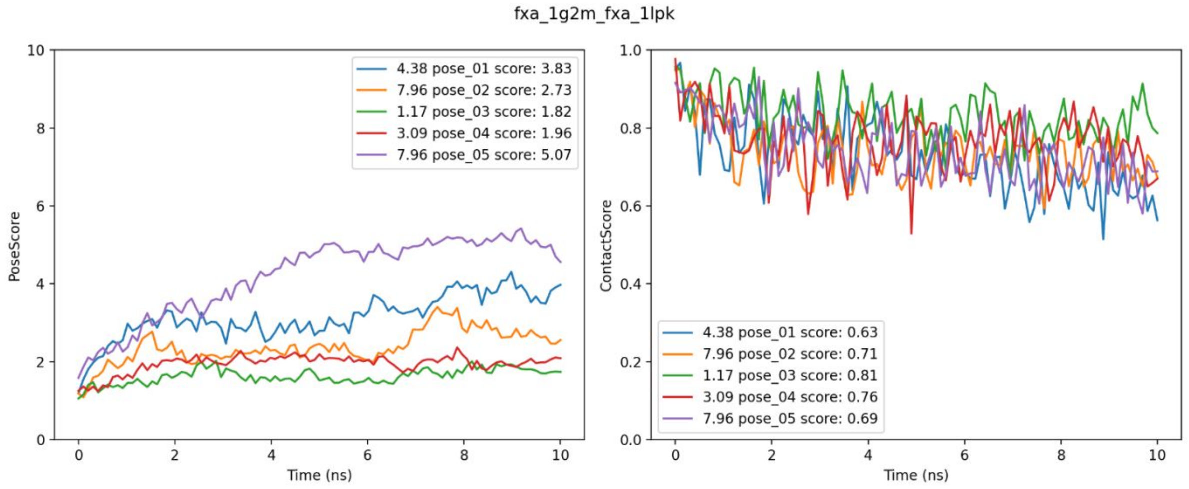
fxa_1g2m_fxa_1lpk
PoseScore
Time (ns)
4.38 pose_01 score: 3.83
7.96 pose_02 score: 2.73
1.17 pose_03 score: 1.82
3.09 pose_04 score: 1.96
7.96 pose_05 score: 5.07
ContactScore
Time (ns)
4.38 pose_01 score: 0.63
7.96 pose_02 score: 0.71
1.17 pose_03 score: 0.81
3.09 pose_04 score: 0.76
7.96 pose_05 score: 0.69

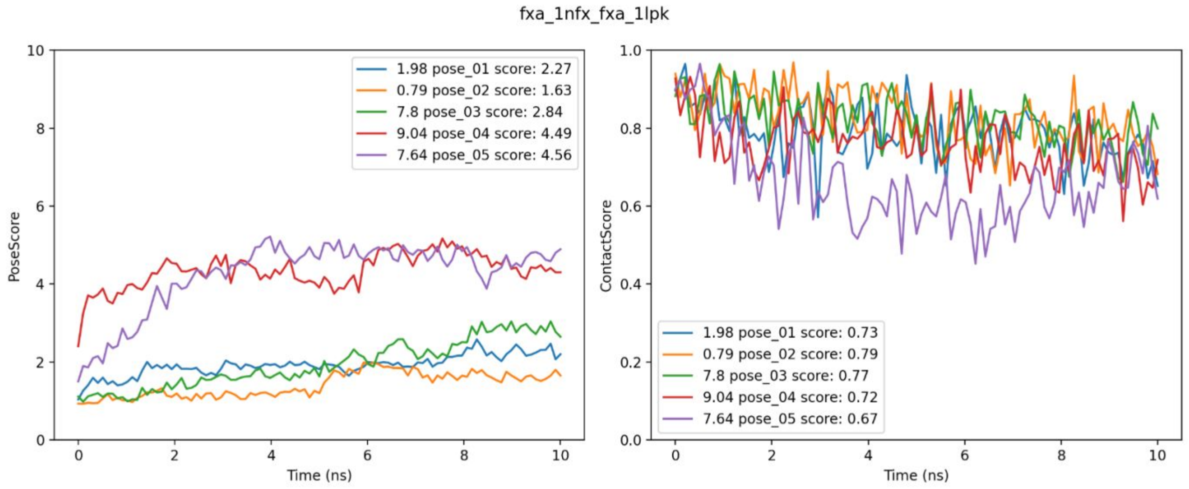
fxa_1nfx_fxa_1lpk
PoseScore
Time (ns)
1.98 pose_01 score: 2.27
0.79 pose_02 score: 1.63
7.8 pose_03 score: 2.84
9.04 pose_04 score: 4.49
7.64 pose_05 score: 4.56
ContactScore
Time (ns)
1.98 pose_01 score: 0.73
0.79 pose_02 score: 0.79
7.8 pose_03 score: 0.77
9.04 pose_04 score: 0.72
7.64 pose_05 score: 0.67

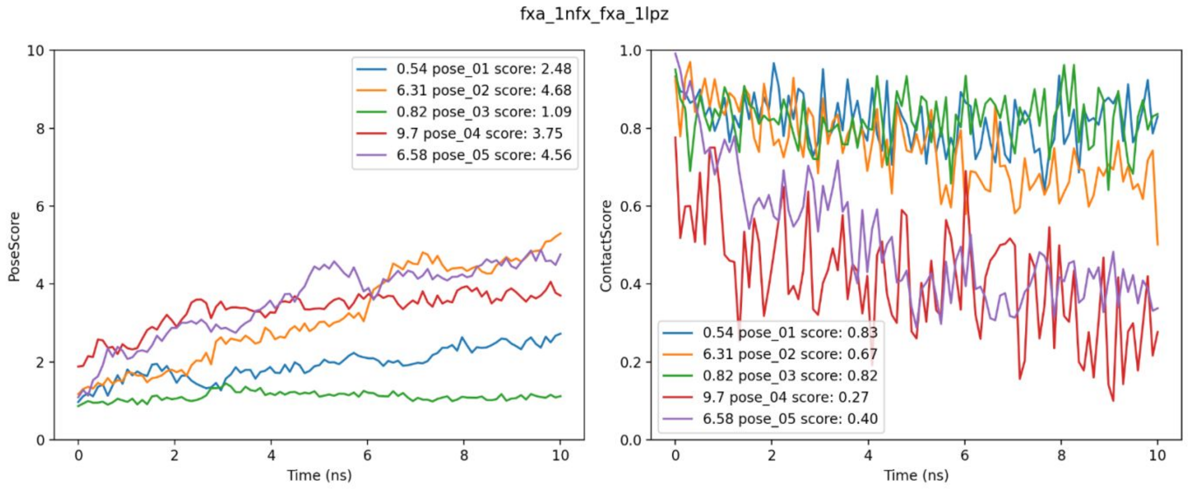
fxa_1nfx_fxa_1lpz
PoseScore
Time (ns)
0.54 pose_01 score: 2.48
6.31 pose_02 score: 4.68
0.82 pose_03 score: 1.09
9.7 pose_04 score: 3.75
6.58 pose_05 score: 4.56
ContactScore
Time (ns)
0.54 pose_01 score: 0.83
6.31 pose_02 score: 0.67
0.82 pose_03 score: 0.82
9.7 pose_04 score: 0.27
6.58 pose_05 score: 0.40

fxa_1nfx_fxa_1lqd

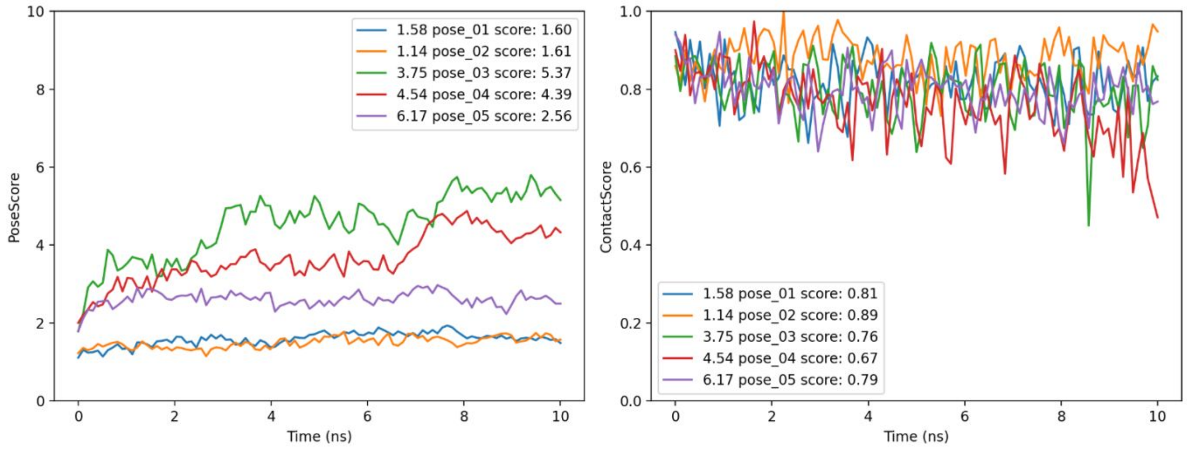

fxa_1xka_fxa_1lqd

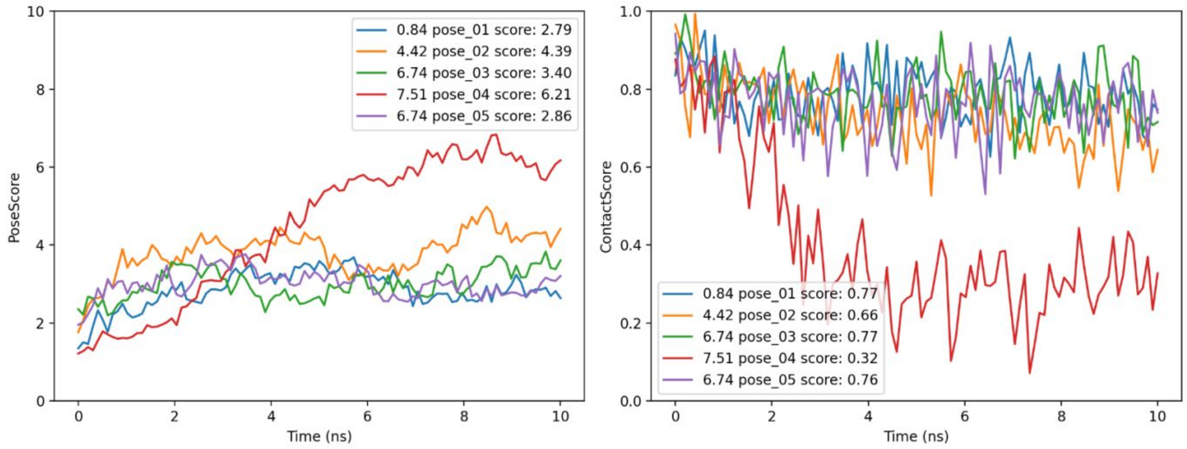

pka_2c1a_pka_1bx6

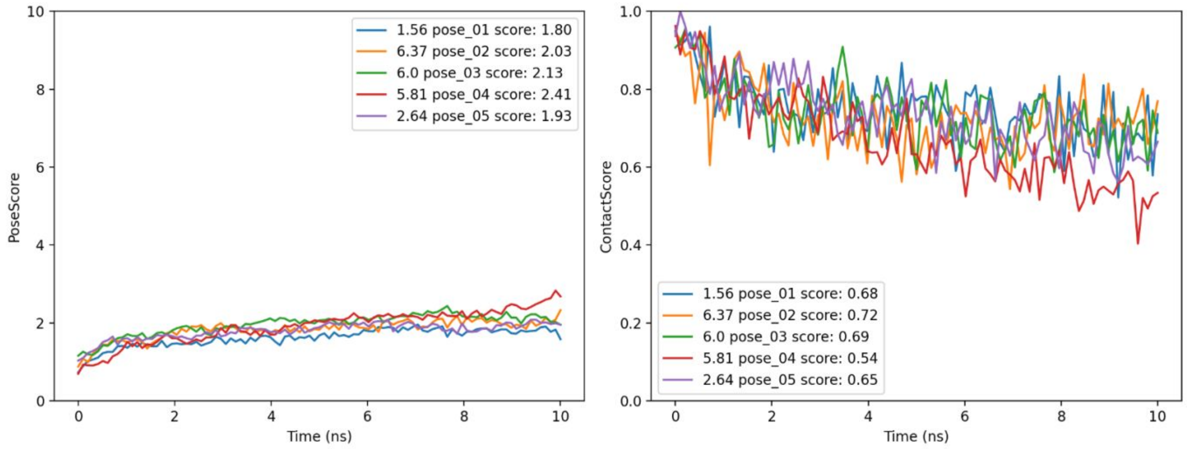

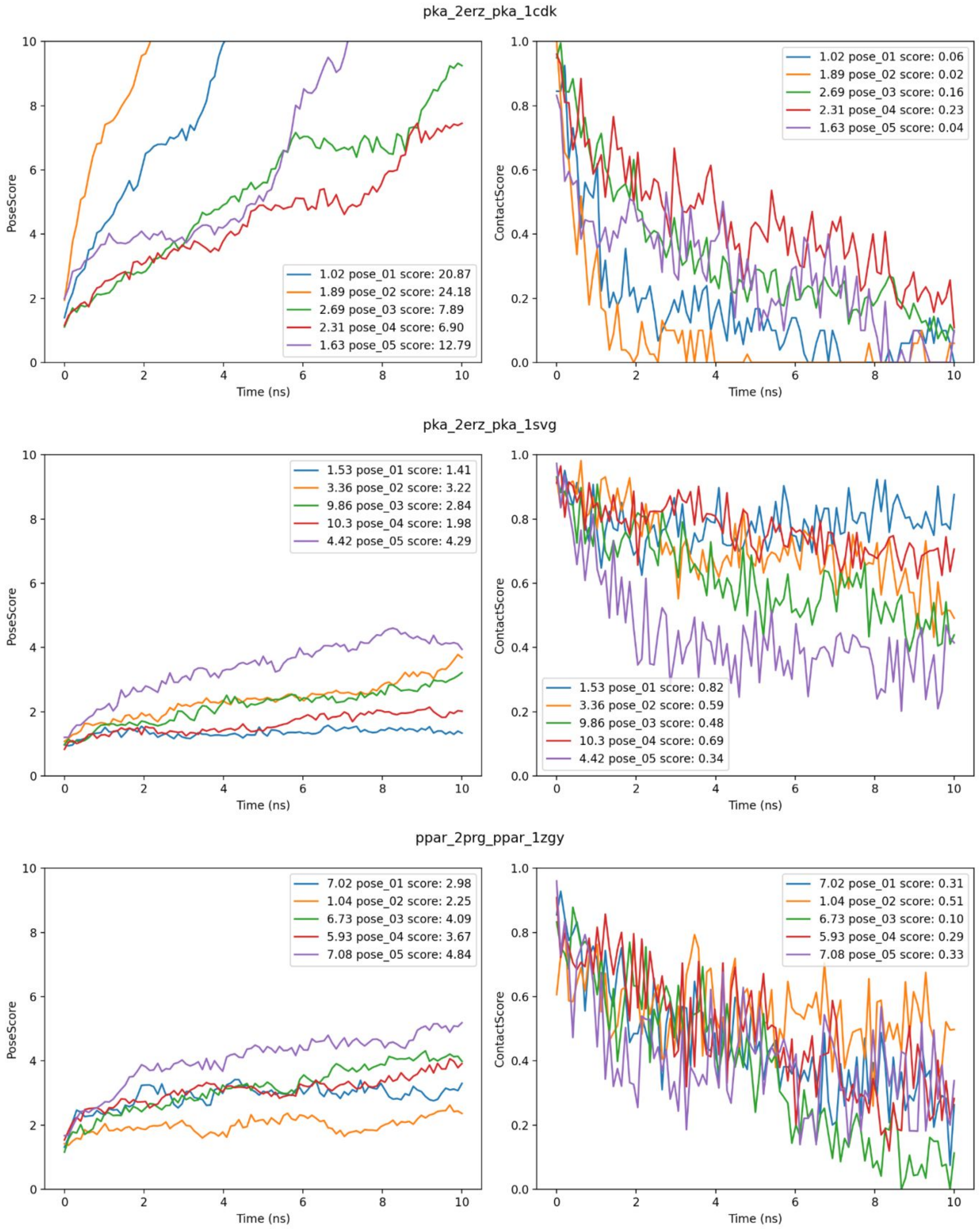
pka_2erz_pka_1cdk
PoseScore
Time (ns)
1.02 pose_01 score: 20.87
1.89 pose_02 score: 24.18
2.69 pose_03 score: 7.89
2.31 pose_04 score: 6.90
1.63 pose_05 score: 12.79
ContactScore
Time (ns)
1.02 pose_01 score: 0.06
1.89 pose_02 score: 0.02
2.69 pose_03 score: 0.16
2.31 pose_04 score: 0.23
1.63 pose_05 score: 0.04
pka_2erz_pka_1svg
PoseScore
Time (ns)
1.53 pose_01 score: 1.41
3.36 pose_02 score: 3.22
9.86 pose_03 score: 2.84
10.3 pose_04 score: 1.98
4.42 pose_05 score: 4.29
ContactScore
Time (ns)
1.53 pose_01 score: 0.82
3.36 pose_02 score: 0.59
9.86 pose_03 score: 0.48
10.3 pose_04 score: 0.69
4.42 pose_05 score: 0.34
ppar_2prg_ppar_1zgy
PoseScore
Time (ns)
7.02 pose_01 score: 2.98
1.04 pose_02 score: 2.25
6.73 pose_03 score: 4.09
5.93 pose_04 score: 3.67
7.08 pose_05 score: 4.84
ContactScore
Time (ns)
7.02 pose_01 score: 0.31
1.04 pose_02 score: 0.51
6.73 pose_03 score: 0.10
5.93 pose_04 score: 0.29
7.08 pose_05 score: 0.33

ppar_2prg_ppar_2ath

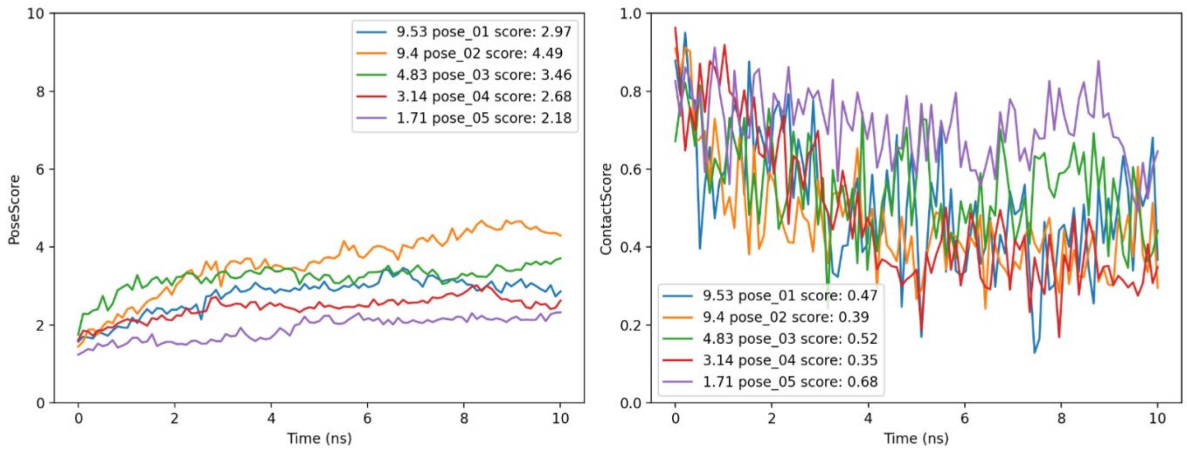

ppar_2prg_ppar_2gtk

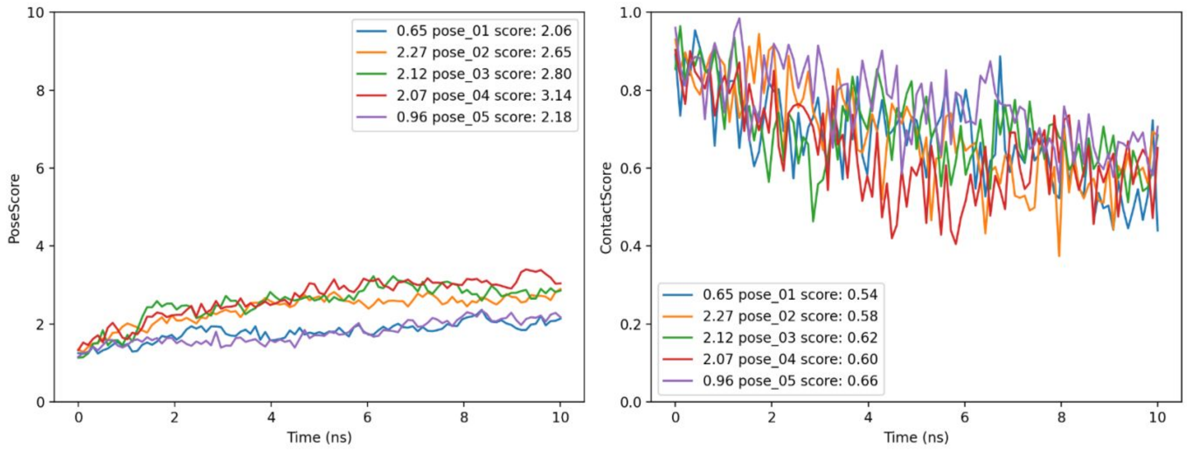

throm_1ae8_throm_1gj4

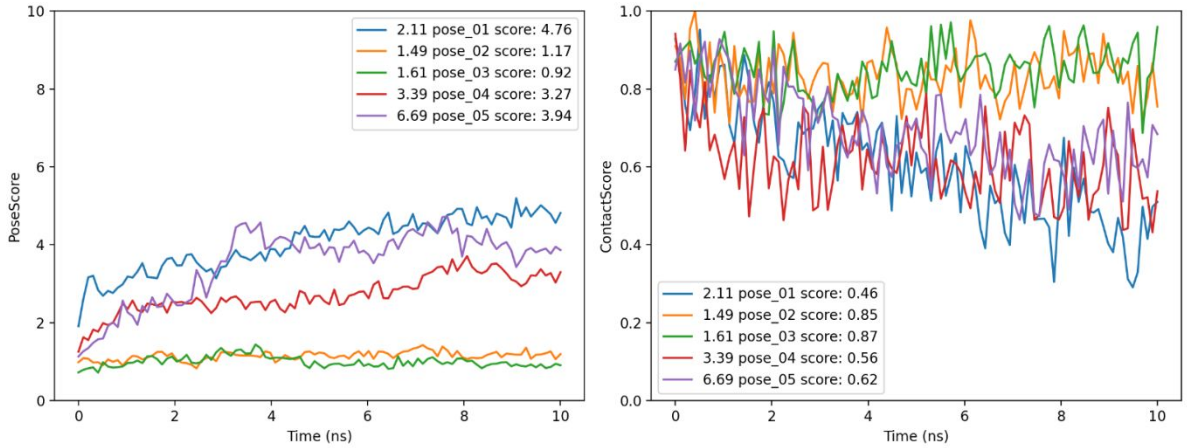

throm_1ae8_throm_1gj5

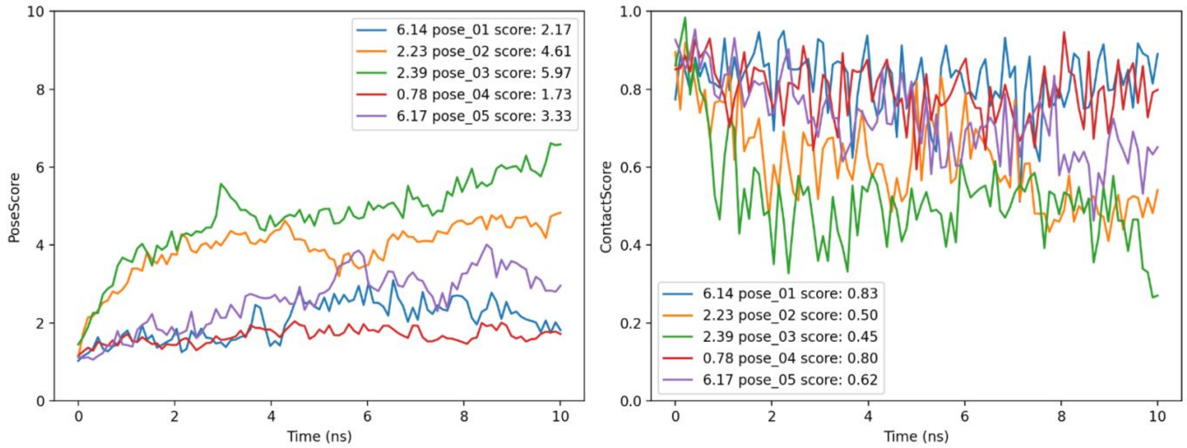

throm_1ae8_throm_1o2g

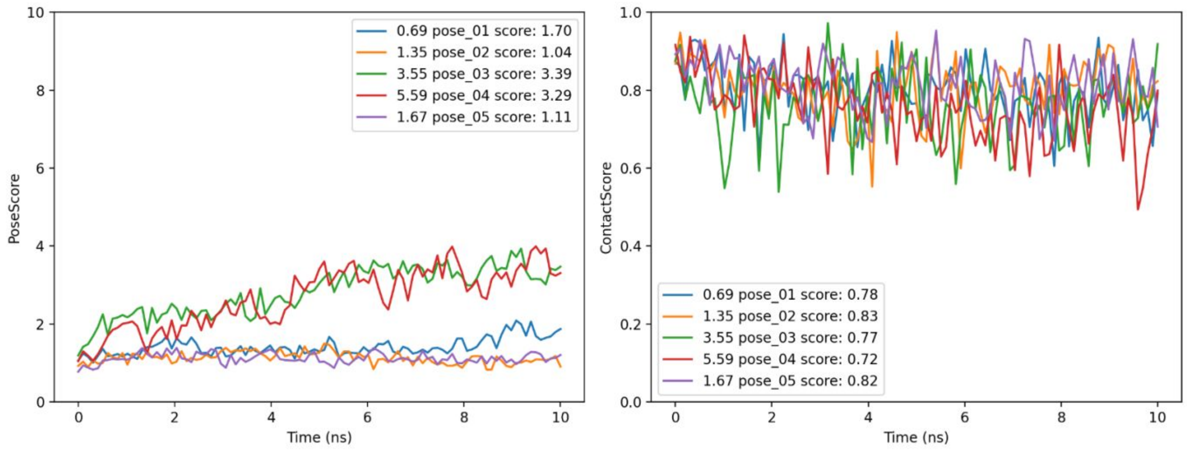

throm_1ae8_throm_1o5g

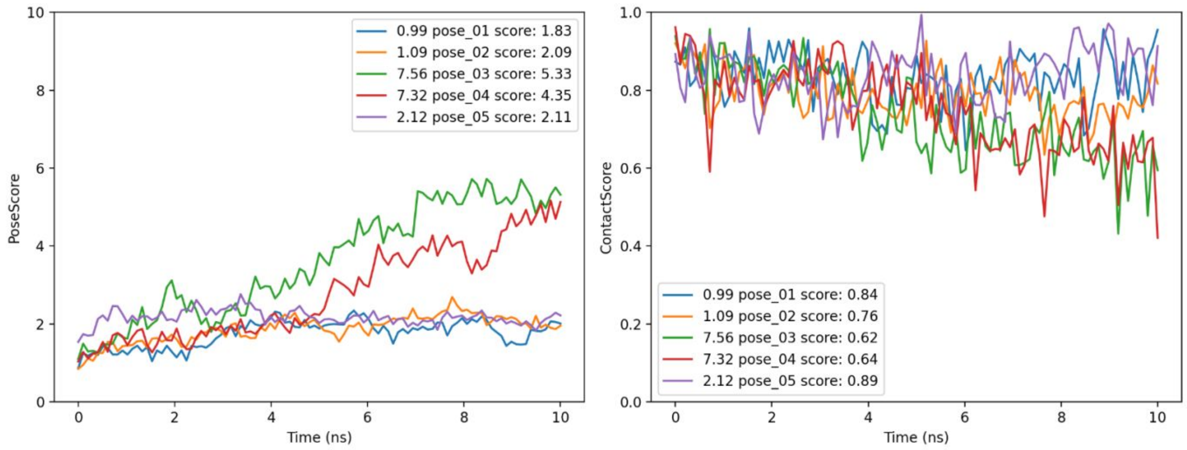

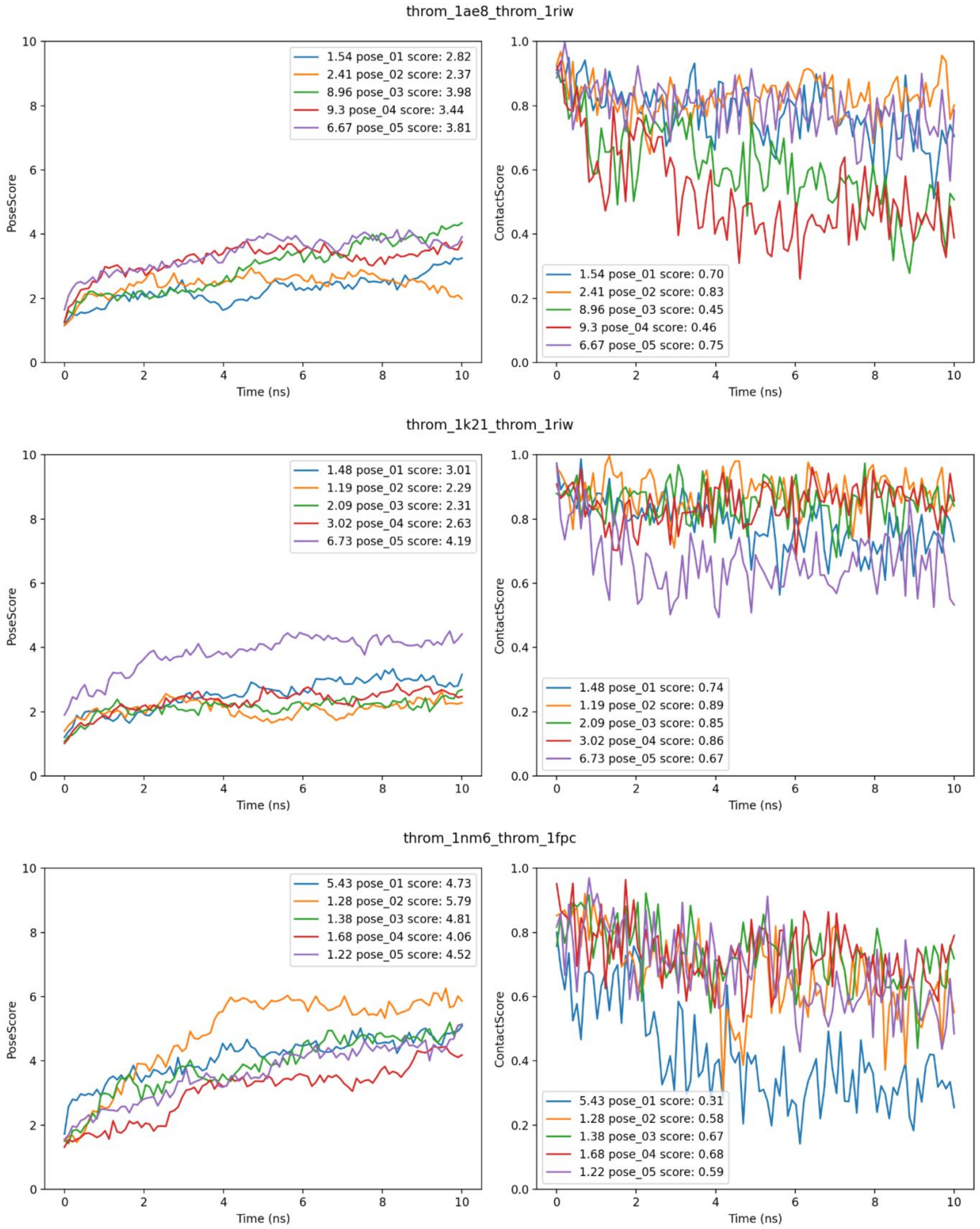

Figure S2. PoseScore and ContactScore plots for each protein-ligand system reported in Tab. S1 that underwent a OpenBPMD+*grand* simulation with 0.3 kcal/mol hill height.

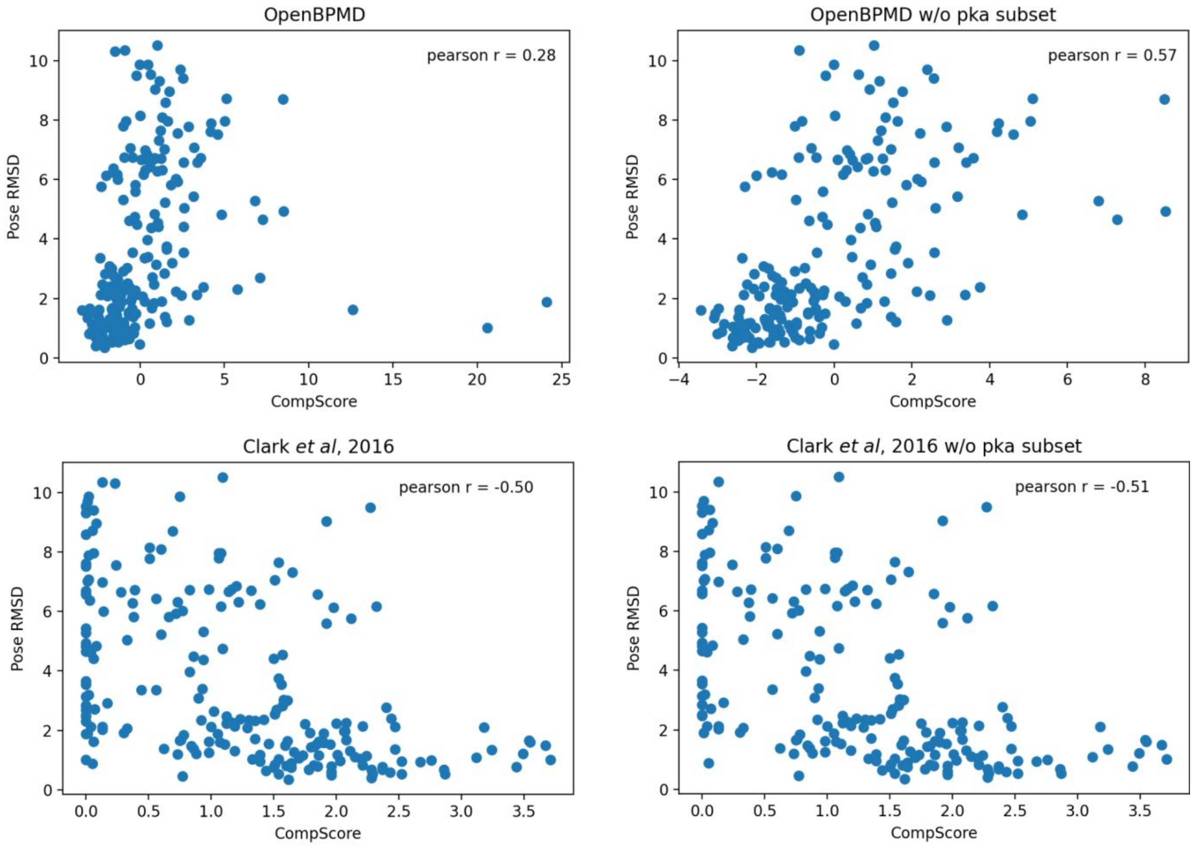

Figure S3. Pearson coefficient correlation analysis between the CompScore and the pose RMSD values. Such analyses were performed for 4 sets of simulations - OpenBPMD+*grand* on the whole Clark *et al.*'s data set[1] (top left); OpenBPMD+*grand* without the pka sub-set (top right); BPMD results from Clark *et al.*[1] (bottom left); BPMD results from Clark *et al.*[1] without the pka sub-set (bottom right). The OpenBPMD+*grand* simulations were performed with a 0.3 kcal/mol hill height (top). For the sake of clarity, in *Clark et al.*[1] the more stable poses had a more positive CompScore score, while in OpenBPMD the more stable poses had a more negative CompScore. This is due to a difference in the formula for combining PoseScore and PersScore. Contrast equation 3 found in the publication by Clark *et al.*[1] and equation 1 in the main text.

Correlation between pose RMSD and CompScore. The pose ranking power of our protocol is its most important feature. While not discussed in the original publication, we were also interested in the correlation between the composite stability score, CompScore, and the pose RMSD, relative to the crystallographic pose. For OpenBPMD, the Pearson *r* correlation between CompScore and Pose RMSD was 0.28, while the results from the original publication give a correlation of -0.50 (the correlation is negative, owing to a difference in the formula for combining PoseScore and PersScore, where the more stable poses had a more positive CompScore score, while in OpenBPMD the more stable poses have a more negative CompScore). As Figure S3 shows, this decrease in the strength of the correlation is driven by a few outliers. All of these low Pose RMSD-high CompScore outliers come from one protein-ligand system, ligand ANP from PDB ID 1cdk docked into a protein kinase A (PKA) receptor structure from PDB ID 2erz. The ligand unbinds from most of the candidate poses during the metadynamics simulations (see Figure S2). The most likely explanation of why the ligand unbinds in our trajectories is the force field. The protocol employed in this work was nearly identical to the one outlined by Clark *et al.*[1] except for the force field. We used the Amber ff14SB protein[2] and GAFF2 ligand force fields,[3] while the work by Clark *et al.*[1] used OPLS3.[4]

When all PKA poses and scores are removed, the correlation between the CompScore and pose RMSD increases from 0.28 to 0.57.

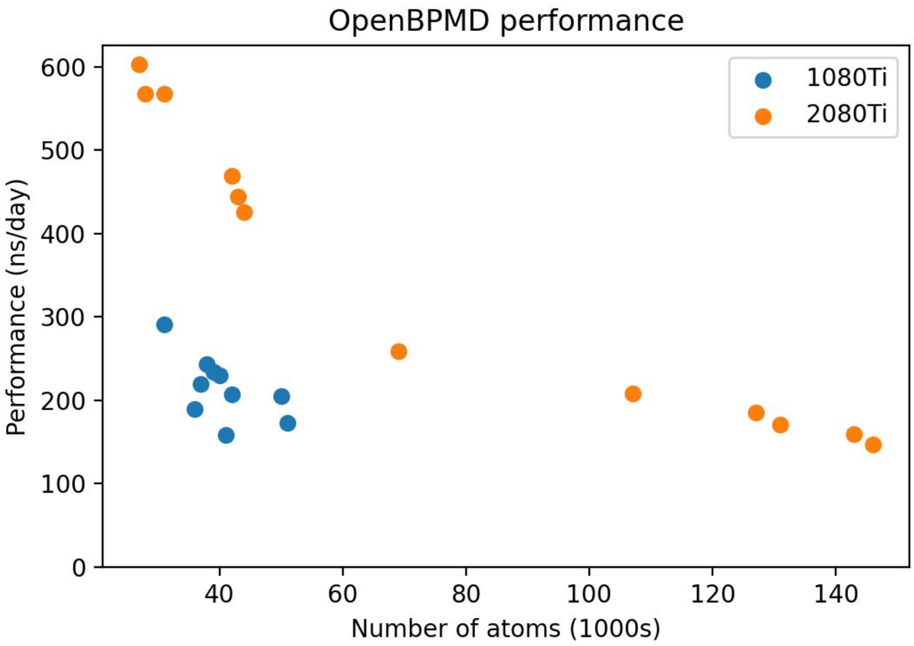

Figure S4. Performance of OpenBPMD on systems of different sizes and on different hardware. All simulations employed a 4 femtosecond timestep and a hydrogen mass repartitioning scheme, with hydrogen masses set to 4 Da. Simulations were run in triclinic boxes. For reference, a typical CDK2 system consisted of around 44 thousand atoms, running at 430 ns/day. If run in series, 10 repeat simulations of such a system would take around 5.5 hours on a single GTX 2080Ti. If run all in parallel, the equivalent would take only 30 minutes. These simulations were run with CUDA version 10.1.

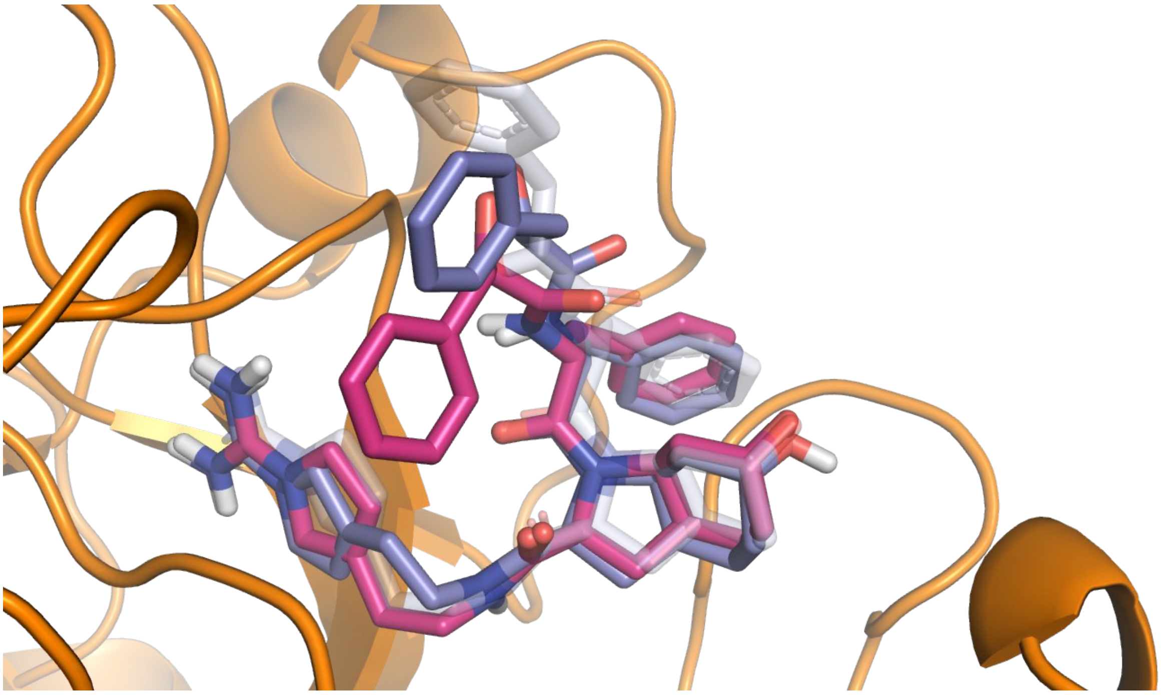

Figure S5. Aligned structures of poses 1 (blue) and 2 (magenta) of a thrombin protein-ligand system with a ligand from PDB ID 1riw and docked into a protein structure from PDB 1ae8. The crystallographic conformation of the ligand OSC found in PDB ID 1riw is shown in transparent white. Pose 2 has a RMSD of 2.41 Å and a CompScore of -1.41, while Pose 1 has a RMSD of 1.54 Å and a CompScore of -0.68. While Pose 2 is above the 2 Å threshold, the source of deviation from the lower RMSD Pose 1 comes from the solvent-exposed and flexible terminal phenyl group (shown towards the top of the image).

The effects of RESP partial charges. To test the effects of the partial charges values on OpenBPMD's predictive power, some of the CDK2 ligands were re-parameterised using the "Restrained Electrostatic Potential" (RESP) approach.[5–7] New RESP partial charges were acquired using an open-source toolkit Psi4.[8] HF/6-31g(d) basis set was used for ligand geometry optimization. The resulting ligand MOL2 files were then parameterised using GAFF2,[3] just as described in the Methods section. We performed OpenBPMD simulations on *grand*-equilibrated protein-ligand structures and using hills with a height of 0.3 kcal/mol. As displayed in Fig. S6, the collected results are even better than the previous calculations, with the binding pose achieved for ligand 8 at an RMSD of ~2 Å with respect to the crystallographic structure.

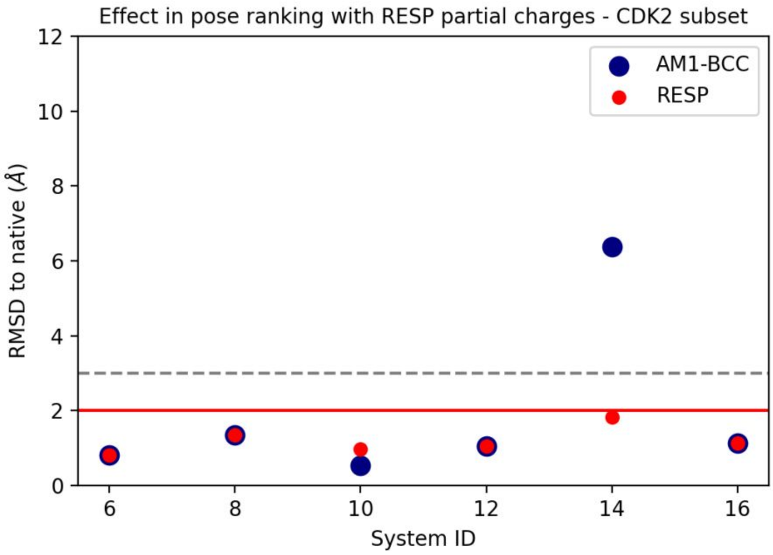

Figure S6. Comparison of OpenBPMD's pose ranking ability for CDK2 subset of ligands with partial charges derived using the AM1-BCC (navy) or the RESP (red) schemes. The percentage of poses correctly ranked < 2 Å RMSD (< 3 Å) threshold went up from 92% (92%) to 100% (100%).

MM-GBSA calculations. Upon reviewer's suggestion, MM-GBSA simulations were run to see if an implicit solvent method could also be useful in ranking ligand poses. Starting structures were the same ones as used in OpenBPMD calculations after *grand* equilibration. A single unbiased MD trajectory was run with OpenMM for 10 ns for each pose, using the same non-bonded parameters as in the main study. Frames were written every 10 ps and the last 900 frames were used in the MM-GBSA calculation. 'igb=2' and 'saltcon=0.15' settings were employed. For an unclear reason, none of the DPP4 protein-ligand structures could be run with the MMPBSA.py script from AmberTools20,[9,10] so the DPP4 binding affinity data was unavailable. As shown in SI Figure S7 below, MM-GBSA is much worse at ranking ligand poses than OpenBPMD. For binding affinities for each pose, consult 'mmgbsa_results.csv'.

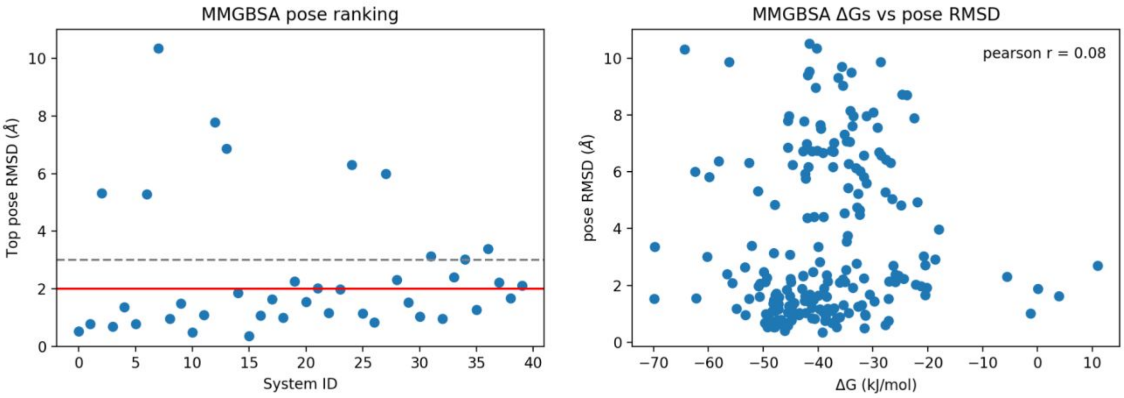

Figure S7. Left - pose ranking of MM-GBSA. It correctly ranked a pose as below the 2 Å (< 3 Å) RMSD threshold 60% of the time (75%). Right - the Pearson correlation between the ligand binding affinity, as determined by MM-GBSA for each pose, and its pose RMSD was just 0.08.

References

(1) Clark, A. J.; Tiwary, P.; Borrelli, K.; Feng, S.; Miller, E. B.; Abel, R.; Friesner, R. A.; Berne, B. J. Prediction of Protein-Ligand Binding Poses via a Combination of Induced Fit Docking and Metadynamics Simulations. *J. Chem. Theory Comput.* **2016**, *12* (6), 2990–2998. https://doi.org/10.1021/acs.jctc.6b00201.

(2) Lindorff-Larsen, K.; Piana, S.; Palmo, K.; Maragakis, P.; Klepeis, J. L.; Dror, R. O.; Shaw, D. E. Improved Side-Chain Torsion Potentials for the Amber Ff99SB Protein Force Field. *Proteins Struct. Funct. Bioinforma.* **2010**, *78* (8), 1950–1958. https://doi.org/10.1002/prot.22711.

(3) Junmei, W.; Romain, W. M.; Cadwell, J. W.; Kollman, P. A.; Case, D. A. Development and Testing of a General Amber Force Field. *J. Comput. Chem.* **2004**, *25*, 1157–1174. https://doi.org/10.1002/jcc.20035.

(4) Harder, E.; Damm, W.; Maple, J.; Wu, C.; Reboul, M.; Xiang, J. Y.; Wang, L.; Lupyan, D.; Dahlgren, M. K.; Knight, J. L.; Kaus, J. W.; Cerutti, D. S.; Krilov, G.; Jorgensen, W. L.; Abel, R.; Friesner, R. A. OPLS3: A Force Field Providing Broad Coverage of Drug-like Small Molecules and Proteins. *J. Chem. Theory Comput.* **2016**, *12* (1), 281–296. https://doi.org/10.1021/acs.jctc.5b00864.

(5) I. Bayly, C.; Cieplak, P.; Cornell, W.; A. Kollman, P. A Well-Behaved Electrostatic Potential Based Method Using Charge Restraints for Deriving Atomic Charges: The RESP Model. *J. Phys. Chem.* **1993**, *97* (40), 10269–10280. https://doi.org/10.1021/j100142a004.

(6) D. Cornell, W.; Cieplak, P.; I. Bayly, C.; A. Kollman, P. Application of RESP Charges to Calculate Conformational Energies, Hydrogen Bond Energies, and Free Energies of Solvation. *J. Am. Chem. Soc.* **1993**, *115* (21), 9620–9631. https://doi.org/10.1021/ja00074a030.

(7) Cieplak, P.; Cornell, W. D.; Bayly, C.; Kollman, P. A. Application of the Multimolecule and Multiconformational RESP Methodology to Biopolymers: Charge Derivation for DNA, RNA, and Proteins. *J. Comput. Chem.* **1995**, *16* (11), 1357–1377. https://doi.org/10.1002/jcc.540161106.

(8) M. Parrish, R.; A. Burns, L.; G. A. Smith, D.; C. Simmonett, A.; Eugene DePrince III, A.; G. Hohenstein, E.; Bozkaya, U.; Yu. Sokolov, A.; Di Remigio, R.; M. Richard, R.; F. Gonthier, J.; M. James, A.; R. McAlexander, H.; Kumar, A.; Saitow, M.; Wang, X.; P. Pritchard, B.; Verma, P.; F. Schaefer III, H.; Patkowski, K.; A. King, R.; F. Valeev, E.; A. Evangelista, F.; M. Turney, J.; Daniel Crawford, T.; David Sherrill, C. Psi4 1.1: An Open-Source Electronic Structure Program Emphasizing Automation, Advanced Libraries, and Interoperability. *J. Chem. Theory Comput.* **2017**, *13* (7), 3185–3197. https://doi.org/10.1021/acs.jctc.7b00174.

(9) R. Miller III, B.; Dwight McGee Jr., T.; M. Swails, J.; Homeyer, N.; Gohlke, H.; E. Roitberg, A. MMPBSA.Py: An Efficient Program for End-State Free Energy Calculations. *J. Chem. Theory Comput.* **2012**, *8* (9), 3314–3321. https://doi.org/10.1021/ct300418h.

(10) Case, D. A.; Ben-Shalom, I. Y.; Brozell, S. R.; Cerutti, D. S.; Cheatham III, T. E.; Cruzeiro, V. W. D.; Darden, T. A.; Duke, R. E.; Ghoreishi, D.; Gilson, M. K.; Gohlke, H.; Goetz, A. W.; Greene, D.; Harris, R.; Homeyer, N.; Izadi, S.; Kovalenko, A.; Kurtzman, T.; Lee, T. S.; LeGrand, S.; Li, P.; Lin, C.; Liu, J.; Luchko, T.; Luo, R.; Mermelstein, D. J.; Merz, K. M.; Miao, Y.; Monard, G.; Nguyen, C.; Nguyen, H.; Omelyan, I.; Onufriev, A.; Pan, F.; Qi, R.; Roe, D. R.; Roitberg, A.; Sagui, C.; Schott-Verdugo, S.; Shen, J.; Simmerling, C. L.; Smith, J.; Salomon-Ferrer, R.; Swails, J.; Walker, R. C.; Wang, J.; Wei, H.; Wolf, R. M.; Wu, X.; Xiao, L.; York, D. M.; Kollman, P. A. AMBER 2018, 2018.
